# Differential benefits of mental training types for attention, compassion, and theory of mind

**DOI:** 10.1016/j.cognition.2019.104039

**Published:** 2020-01

**Authors:** Fynn-Mathis Trautwein, Philipp Kanske, Anne Böckler, Tania Singer

**Affiliations:** a Edmont J. Safra Brain Research Center, University of Haifa, Haifa, Israel; b Clinical Psychology and Behavioral Neuroscience, Technische Universität Dresden, Chemnitzer Straße 46, 01187 Dresden, Germany; c Department of Psychology, Würzburg University, Röntgenring 11, 97070 Würzburg, Germany; d Max Planck Society, Social Neuroscience Lab, Berlin, Germany; e Max Planck Institute for Human Cognitive and Brain Sciences, Leipzig, Germany

**Keywords:** Attention, Theory of mind, Compassion, Mindfulness meditation, Mental training

## Abstract

Mindfulness- and, more generally, meditation-based interventions increasingly gain popularity, effectively promoting cognitive, affective, and social capacities. It is unclear, however, if different types of practice have the same or specific effects on mental functioning. Here we tested three consecutive three-month training modules aimed at cultivating either attention, socio-affective qualities (such as compassion), or socio-cognitive skills (such as theory of mind), in three training cohorts and a retest control cohort (*N* = 332). While attentional performance improved most consistently after attention training, compassion increased most after socio-affective training and theory of mind partially improved after socio-cognitive training. These results show that specific mental training practices are needed to induce plasticity in different domains of mental functioning, providing a foundation for evidence-based development of more targeted interventions adapted to the needs of different education, labor, and health settings.

## 1 Introduction

In a more and more complex and interconnected world—with constant exposure to multi-channel online stimulation, global competition for limited resources, and penetration of socio-cultural borders—the question if and how human capacities such as attention and social and emotional intelligence can be cultivated has become increasingly salient. Here, meditation-based mental training might represent an effective means to induce plasticity in relevant cognitive, affective, and social functions (Goyal et al., 2014, Sedlmeier et al., 2012, Tang et al., 2015). However, previous research focused on interventions that integrate a range of different contemplative practices, such as on the well-known mindfulness-based stress reduction program (MBSR) (Kabat-Zinn, 1990), and mostly lacked the direct comparison with other meditation-based control conditions. Therefore, it remains unclear whether different types of practice, pursuing different aims (Dahl, Lutz, & Davidson, 2015), can induce plasticity in distinct mental functions.

The typical 8-week MBSR program, for instance, emphasizes practices that involve attention to present-moment awareness of perception and interoception, but also includes components directed at awareness of thoughts and emotions (Kabat-Zinn, 1990). Moreover, other meditation-based programs focus on the cultivation of socio-affective capacities such as compassion and loving-kindness, but also include practices that address mindfulness and attention (Jazaieri et al., 2013, Mascaro et al., 2013, Neff and Germer, 2013). Thus, the specific fingerprints of these mental practices are still poorly understood despite the fact that future evidence-based implementation of interventions adapted to specific needs of health, labor, or education contexts will require knowledge of their outcome specificity.

Recent theoretical accounts have suggested taxonomies of contemplative practices based on characteristics of the involved method, states, and suspected outcomes (Dahl et al., 2015, Dahl et al., 2016, Engen and Singer, 2015a, Nash and Newberg, 2013). For example, Dahl et al. (2015) distinguished attentional, constructive, and deconstructive practices. While practices in the attentional family assumedly enhance attention, constructive and deconstructive practices are thought to favorably alter patterns of cognition and emotion. To date, however, there is little evidence for such specificity of mental training effects. Furthermore, even though an increasing number of studies now use active control conditions (Allen et al., 2012, Weng et al., 2013), these active controls usually do not involve mental training.

**Fig. 1:**
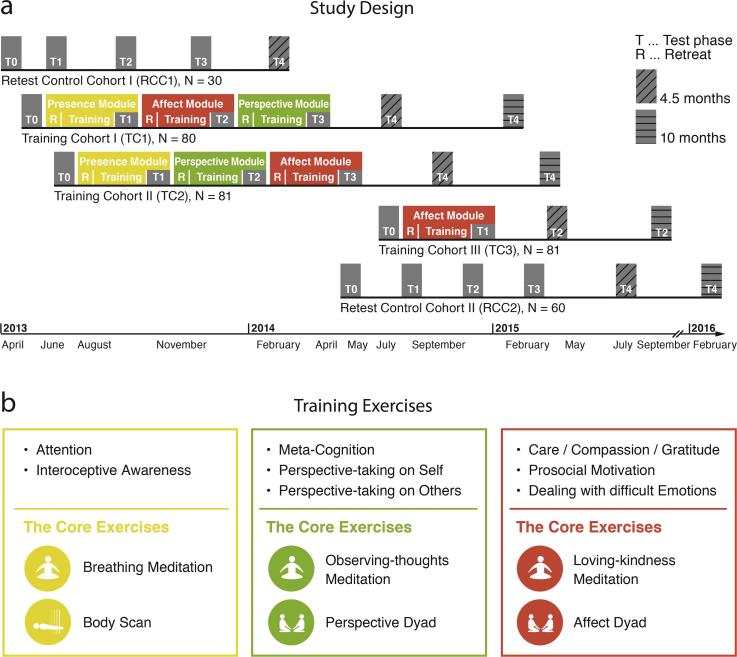
**Study design.** (**a**) Timeline of training (colored areas) and data collection (gray areas) for the training and retest control cohorts. The modules were completed in different orders, allowing using these as active control conditions for each other. For practical testing reasons, all cohorts proceeded in a shifted manner, and retest participants were split into two cohorts but are jointly analyzed. Retest cohorts completed the measurements without any training. The full *ReSource* Design as shown in the figure also included follow-up assessments; however, these are not included in the present investigation. (**b**) Illustration of core exercises of the three modules: Presence (yellow), Affect (red), Perspective (green). Please refer to the Material and Methods section for details. Figures were adapted from (Singer et al., 2016). (For interpretation of the references to colour in this figure legend, the reader is referred to the web version of this article.)

Stringent evidence for effectiveness and specificity of training induced plasticity of mental functions such as attention, social emotions, and social cognition requires a study design that implements different types of contemplative practices within structurally equivalent intervention conditions (e.g. with respect to setting, teachers, and amount of training). To this end, we designed a large-scale longitudinal mental training study, the ReSource Project (Singer et al., 2016), implementing three consecutive three-month mental training modules (Presence, Affect, Perspective) consisting of distinct types of contemplative practices and targeting attentional-, socio-cognitive, or socio-affective skills (Fig. 1). The Presence Module focused on cultivating present-moment attention and interoceptive awareness through exercises (e.g. Breathing Meditation and Body Scan) typically employed in other mindfulness-based interventions such as MBSR (Kabat-Zinn, 1990). The Affect Module focused on cultivating affective qualities of care, gratitude, and loving-kindness, as well as dealing with difficult emotions through acceptance and increasing prosocial motivation. The Perspective Module focused on improving metacognitive awareness of thoughts and perspective taking on self and others (the latter also referred to as mentalizing or theory of mind; ToM) (Brüne and Brüne-Cohrs, 2006, Mitchell, 2009, Wimmer and Perner, 1983).

Awareness of present-moment experience and stability of attention—as cultivated by the Presence Module—are often regarded as fundamental for contemplative practice. Therefore, the ReSource training program started with the Presence Module for both main cohorts. Subsequently, these cohorts completed the Affect Module and the Perspective Module in counterbalanced orders, allowing for sensitive statistical assessment of within-subject differential effects. In addition, to test for specific effects elicited by the Presence Module, we added another active control group (TC3) that underwent only the three-month Affect training without a preceding Presence Module. Contributing to previous debates about the relationship between mindfulness and prosocial qualities such as compassion (Brown and Ryan, 2004, Shapiro et al., 2006, Grossman, 2008), adding an Affect only group allowed us to address related questions, such as whether explicit compassion-based training alone effectively enhances compassion (Neff & Germer, 2013), and whether (previous) training of mindfulness in terms of present-moment awareness (as implemented in the Presence Module) is beneficial to this end. Additional enrollment of a further cohort only receiving the Perspective training was not realizable due to monetary and time constraints. In sum, for each type of mental training (i.e. training module), the design includes at least one high level active control condition as well as the comparison to retest, thereby enabling stringent assessment of differential training effects.

**Fig. 2:**
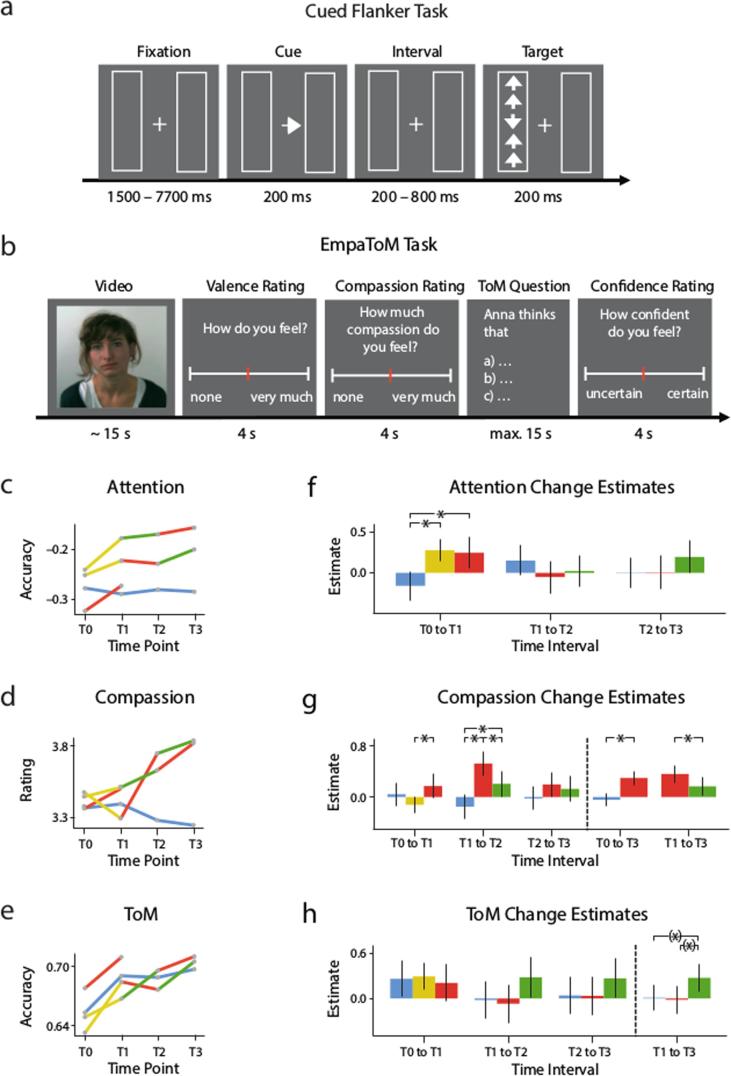
**Illustration of behavioral tasks, descriptive data, and model estimates.** (**a**) Illustration of one trial of the cued flanker task that was used to assess attention. (**b**) Illustration of one trial of the EmpaToM task that was used to assess trial-wise experience of compassion and accuracy of mental state inference (i.e. ToM). Note that this simplified illustration omits fixation periods between the screens and the name of the speaker presented in the beginning of each trial. For details of the tasks, please refer to the methods section. (**c-e**) Descriptive plots of mean values for attention (difference of correct response proportions in the reorienting and conflict condition minus baseline condition), compassion (mean ratings on a scale from 0 to 6) and ToM (proportion of correct responses in ToM questions) per time point and group. Note that differences between groups at T0 were not significant (F-Test p-values all >.11). The mean values of each individual were used to calculate change scores for each available pair of two consecutive time points, which were used to estimate effects for retest and the three training modules shown in (**f-h**). In panels (**g**) and (**h**), to the right of the dashed lines, estimates are averaged across time intervals as was done to test the main hypotheses for these two measures. Error bars in (**f-h**) indicate 95% confidence intervals.

Here, we focused on three target measures, namely behavioral markers of attention (Fig. 2a), compassion, and ToM (Fig. 2b), as we expected the three modules to have differential effects on these specific outcomes. The ReSource project also includes a range of other questionnaire, physiological, and behavioral measures targeting related constructs (cf. Singer et al., 2016). The present study focuses on socio-affective and socio-cognitive markers assessed in a single task, the EmpaToM, that was explicitly developed for the ReSource Project in order to allow measurement of core outcomes of the two “social” training modules, Affect and Perspective, with stimulus material enabling repeated measurement at up to four measurement timepoints (Kanske et al., 2016, Kanske et al., 2015). These measures are complemented by an attention marker (Trautwein, Singer, & Kanske, 2016) which was selected as an outcome of the Presence Module because: (1) In parallel to compassion and ToM being directly linked to the Affect and Perspective modules, attention is a core hypothesized outcome of the Presence Module (see Fig. 1). (2) It is measured at a similar level of granularity in terms of trial-wise performance and was assessed at the same day and within the same (scanning) environment as the EmpaToM task; (3) It therefore is the measure best comparable to the compassion and ToM markers.

We expected the Presence Module to improve attention based on both (a) its focus on increasing present-moment attention and (b) previously reported effects of similar mindfulness interventions on attention (Allen et al., 2012, Slagter et al., 2007, Tang et al., 2007). An open question is, however, whether attention is also enhanced by other types of mental practices, for example those focused on loving-kindness (Salzberg, 1995), that do also (implicitly) demand self-regulation of attention.

Second, based on its focus on cultivating social emotions, we expected the Affect Module to particularly increase compassion, defined as feelings of warmth, care and concern (Singer & Klimecki, 2014). Accordingly, previous intervention studies on compassion and loving-kindness meditation provided evidence for increased positive affect and concomitant activation increases in neuronal networks associated with positive emotions, affiliation, and care (Engen and Singer, 2015b, Klimecki et al., 2013, Klimecki et al., 2014, Kok and Fredrickson, 2010). Moreover, it has been debated how exactly ethical-motivational qualities such as compassion are linked with mindfulness (Brown and Ryan, 2004, Grossman, 2008, Neff and Germer, 2013); and thus it is unclear whether such qualities are cascade-like outcomes of present-moment awareness and attention focused practices as implemented in the Presence Module—and would therefore also be enhanced by this module alone. Third, we expected the Perspective Module to be particularly efficient in enhancing ToM. The module includes practices explicitly targeting this intersubjective skill and is based on evidence of distinct neural networks supporting ToM vs. empathy and compassion (Kanske et al., 2016, Kanske et al., 2015, Singer, 2012, Valk et al., 2017, Valk et al., 2017). While some studies have associated mindfulness with a shift in the perspective on one’s own experience (Lebois et al., 2015), to date, little is known about whether (and which) contemplative practices foster perspective taking on others. More general meditation effects on social cognition have been assessed using the Reading the Mind in the Eyes Test—a task that has recently been shown to measure emotion recognition, rather than ToM (Oakley, Brewer, Bird, & Catmur, 2016)—however with heterogeneous results (Mascaro et al., 2013, Melloni et al., 2013). Thus, it remains unknown whether and which type of mental training can actually improve ToM accuracy. Such evidence would be highly informative for the design of interventions for a range of clinical conditions with ToM impairments (Brüne & Brüne-Cohrs, 2006).

Finally, in addition to these comparisons between training modules, within each module, we compared the effects on the the three behavioral measures (attention, compassion, ToM), thus testing whether each module had stronger or even unique effects on the respective outcome measure.

## 2 Material and methods

### 2.1 Experimental design

The design of the large-scale 9-month mental training study, the ReSource Project (Singer et al., 2016), allowed us to assess differential effects of three 3-month training modules (Presence, Affect, Perspective) in a highly conservative and well controlled design by comparing pre-post effects of each module against a retest cohort and against the other training modules both in a between and within subject fashion (Fig. 1a). Participants were assigned to one of three training cohorts (TC1, TC2, TC3) or a retest control cohort (RCC). Participants from a first recruitment were assigned to TC1, TC2, or RCC; a second recruitment was done for TC3 and additional RCC participants. Assignment was done using a bootstrapping process which ensured that all cohorts were matched for age, gender, marital status, income, IQ, and a number of personality trait questionnaires (*p* for all comparisons > .1; see Singer et al., 2016). Two training cohorts (TC1 and TC2) completed all three training modules in different orders thus functioning as active control groups for each other, while TC3 only did the Affect Module to serve as active control for the Presence Module. Each module lasted for about 3 months (nearly 13 weeks) and all measures were assessed prior to training and during the last 5 weeks of each module. For each timepoint, assessments included 8 structural and functional MRI paradigms, 4 virtual reality scenarios, 10 computer paradigms, 5 economical games, 5 measures of autonomic nervous system functioning, 9 biological measures, 49 questionnaires and experience sampling (see Singer et al., 2016 for details).

### 2.2 Participants

Within two recruitment waves, a total of *N* = 332 healthy participants (197 female; mean age = 40.74, SD = 9.24; age range = 20–55) were selected for and agreed to participate in the study (see Singer et al., 2016 for a detailed description of the multi-step recruitment and screening procedure and characteristics of the final sample for each cohort). Note that the third training cohort (TC3) and a part of the retest cohort (RCC) were added to the study in a second recruitment wave. This was necessary (1) due to practical limitations (cohorts had to start at different time points due to capacity restrictions of the recruitment team, the scanner and other testing facilities) and (2) availability of enough resources for TC3 was only guaranteed at a later point, which then allowed realization of this active control condition for the Presence training. Since the study involves a large range of outcomes, the sample size was determined prior to recruitment based on practical considerations and exceeds previously used sample sizes (Tang et al., 2015). From the first recruitment wave, 191 participants were selected and assigned to the RCC (*N* = 30), TC1 (*N* = 80), or TC2 (*N* = 81). From the second wave, 141 participants were selected and assigned to the RCC (*N* = 60) or to TC3 (*N* = 81). Across all four time points throughout the entire study, 78% (attention task) and 85% (EmpaToM) of the data were available and usable for analysis. As detailed in Table S1, missingness occurred due to study dropout/exclusion (6%), partial dropout/exclusion from MRI experiments (4%), technical, health, or scheduling issues at individual assessments (4% attention task, 5% EmpaToM), and poor or incorrect task performance (9%, only for the attention task). Criteria for poor or incorrect task performance were the same as in a previous study using baseline data from the attention task (Trautwein et al., 2016), that is, datasets with error-rates exceeding 50% in one of the experimental blocks or with a percentage of misses above 12.5% were excluded.

Finally, since our analysis focused on change scores (see below), the sample of the analysis was restricted to participants and time intervals where both pre- and post-scores were available (see Supplemental Material, Table S1 shows the number of datasets available for each time point, while Table S2 shows the number of change scores that could be calculated from these).

All participants gave informed consent prior to participation and the study was approved by the Research Ethics Committee of the University of Leipzig, number 376/12-ff and the Research Ethics Committee of the Humboldt University in Berlin, numbers 2013-02, 2013-29, and 2014-10. The study was registered with the Protocol Registration System of ClinicalTrials.gov under the title “Plasticity of the Compassionate Brain” with the ClinicalTrials.gov Identifier: NCT01833104. The measures reported here are listed as primary outcomes under “functional magnetic resonance imaging (fMRI) measure: Cued flanker task (CueFla), an attention and orienting task” and “functional magnetic resonance imaging (fMRI) measure: a Theory of Mind and Social Cognition task (the EmpaToM)”. Note that the descriptions were updated during the course of the study to assure consistent terminology and accomodate the addition of TC3.

### 2.3 Training modules of the ReSource study

All three ReSource training modules (Presence, Affect, Perspective) lasted for 3 months, beginning with a 3-day intensive retreat, followed by 13 weekly group sessions with the teachers as well as daily home practice facilitated by a custom-made internet platform and smartphone applications providing audio streams for the guided meditations and an interface for the dyadic exercises. This also allowed for daily recording of practice time and duration and compliance to the practice (see Singer et al., 2016 for details). During the retreat, participants were introduced to the module's topics and the respective core exercises (see Fig. 1b). The 2-h weekly sessions with teachers included discussion of training challenges and effects, practice of the two core exercises, and introduction of new contemplative practices aiming at cultivating the respective processes of each module (see Fig. 1b). The last 5 weeks of each module were used to consolidate previous topics and no new topics were introduced.

The core exercises of the Presence Module, practiced repeatedly during the retreat, in the weekly sessions, and daily home practice (instruction was to practice at least five times per week), were Breathing Meditation and Body Scan (Kabat-Zinn, 1990). The basic instruction for Breathing Meditation was to focus attention on sensations of breathing, and to refocus attention whenever it wandered. The Body Scan involved focusing on various parts of the body in a systematic fashion (e.g. from the toes to the head) while paying close attention to sensations occurring in these parts of the body. Additional exercises of the Presence Module, which were practiced during the Retreat or weekly sessions, were walking meditation, meditations on vision, sound, taste and open presence. These practices require a deliberate focus of attention on certain aspects of present moment-to-moment experience, monitoring of distractions, and reorienting towards the object of attention in the meditation, be it the breath, a sound, or a visual object.

The core exercises of the Affect Module were Loving-kindness Meditation (Salzberg, 1995) and the so-called Affect Dyad. For Loving-kindness Meditation, participants were first introduced to various ways of connecting with the feeling and motivation of love and care, such as imagining a baby, a cute animal, a close benevolent other, a place of safety and comfort, or focusing on feelings of warmth in the body. These feelings can then be directed towards oneself and others. The typical instruction for the Loving-kindness Meditation was to start by imagining oneself and then a benefactor, where such feelings might arise naturally, and then to extend these feelings of loving-kindness and good wishes to oneself and then the benefactor. Over the course of one or several meditation sessions, participants were then asked to extend these feelings to others to whom they felt neutral, people they had difficulties with, and ultimately all humans and sentient beings. To stabilize and foster experiences of loving-kindness, participants were instructed to mentally repeat phrases such as “May you be happy,” “May you be healthy,” “May you be safe,” and “May you live with ease.”

The Affect Dyad is a partner exercise, that was done face to face during the retreat and weekly sessions, and through a web or smartphone based application during the daily practice at home. During this exercise, participants contemplated situations from their last day: those which they experienced as difficult and situations for which they were grateful. One participant then listened attentively to what the speaker had to say without giving verbal or non-verbal feedback, thus cultivating empathic listening. The speaker remembered the situation and how it felt and focused on the immediate subjective affective and bodily experience without engaging in abstract reasoning or interpretation. After a first run, roles were switched. This contemplative dialogue allows cultivation of empathic listening in the listener and observation of difficult emotions and their effect on the body as well as development of gratitude and positive affect in the speaker. Additional elements of the Affect Module were exploration of emotions in an attitude of acceptance and care, a guided meditation that contrasts empathy and compassion and teaches participants how to transform an empathic into a loving compassionate response when confronted with suffering of others (Klimecki et al., 2014), forgiveness meditation, and development of self-compassion (Neff & Germer, 2013). Thus, all of the exercises focused on developing an accepting, kind, and compassionate stance towards oneself and others.

The core exercises of the Perspective Module were Observing-thoughts Meditation and Perspective Dyad. In Observing-thoughts Meditation the objective is to observe thoughts as mental events or natural phenomena and not as direct representations of reality. In the initial phase of the practice, this was supported by the labeling of thoughts using opposite poles such as me/other, past/future, positive/negative, or more generic labels such as “judging” and “thinking”. Later in the program, participants were instructed to just observe the coming and going of thoughts without getting involved in them.

The Perspective Dyad is a partner exercise with a structure similar to the Affect Dyad. This exercise was partly based on the Internal Family System approach by Schwartz and colleagues (Holmes, 2007, Schwartz, 1997) and partly on previous theoretical accounts distinguishing between socio-affective (e.g., compassion and empathy) and socio-cognitive (e.g., ToM) routes of social cognition (see Singer, 2006, Singer, 2012 for reviews). For this perspective taking exercise on self and others, participants were first introduced to the concept of inner parts, personality-trait-like patterns of cognition, emotion and behavioral tendencies which dominate in certain situations and shape experience and behavior (Holmes, 2007). During the retreat and throughout the course, participants were supported in identifying inner parts. In the Perspective Dyad, the speaker described a situation from the last day from the perspective of one of his/her inner parts, that is, how the experience might have been if a certain inner part had been dominant in that situation. The counterpart listened attentively without giving verbal or non-verbal feedback and tried to find out from which inner part the speaker was recounting the situation, that is, the listener had to engage in cognitive perspective taking on the other to find out “who is speaking” and to infer which needs, desires, and belief system the other had. The speakers in turn needed to take a meta-perspective onto their own self-related aspects and to de-couple from a lived and experienced reality. Additional elements of the Perspective Module were exercises in which participants needed to take the perspective of people with whom they encounter difficulties in their daily lives, reflected on the central role that thoughts play in our lives, how these might differ for others, and why understanding them differs from approving their behavior. This description of the training protocol was adapted from Singer et al. (2016).

### 2.4 Behavioral outcome measures

As a behavioral marker for attentional performance we used a cued flanker task (Eriksen & Eriksen, 1974; Posner, 1980; Trautwein et al., 2016), whereas compassion and ToM performance were assessed in the EmpaToM video task (Kanske et al., 2015). Both measures were assessed on the same day during functional magnetic resonance imaging (fMRI). Note that respective imaging data will be reported in separate publications.

The cued flanker task assesses two main attention functions, executive control and stimulus driven reorienting of attention, both in isolated and in concurrent demand conditions. Specifically, the task combines a flanker-target conflict (Eriksen and Eriksen, 1974, Fan et al., 2002) with spatial cueing of the target location (Corbetta et al., 2000, Posner, 1980) and thus allows assessing shared and isolated resources of these hallmarks of attention (Petersen and Posner, 2012, Trautwein et al., 2016).

As previously reported on the baseline data of the present study (Trautwein et al., 2016), concurrent demand of stimulus-driven reorienting and executive control of attention leads to over-additive increases in response costs, indicating that both functions rely on a common bottleneck or a general attentional capacity. To test for improvements in attention in the most comprehensive way and under the most challenging conditions, our analysis focused on the concurrent demand condition. For a more detailed picture, we also tested changes in the isolated markers of both functions.

The 15-minute task presented 240 trials with the following structure (see Fig. 2a): After a random fixation period (1500, 1700, 2100, 2900, 4500, or 7700 ms), a central arrow cue appeared for 200 ms indicating the position of the target stimulus. After a random interval (200, 500, or 800 ms), five arrows appeared at the cued location (valid cue condition, 80% of the trials) or at the uncued location (invalid cue condition, 20% of the trials). Participants were instructed to press one of two buttons depending on the direction of the middle arrow (index finger of the right hand for upward arrows and middle finger of the right hand for downward arrows). In half of the trials the middle arrow was flanked by congruent arrows pointing in the same direction (congruent target condition), and in the other half by arrows pointing in the opposite direction (incongruent target condition). After each third of the task, two successive visual analogue scales assessing task focus and task unrelated thoughts were presented for eight seconds each. Prior to the measurement session, participants were familiarized with the task in a short training session (30 trials).

The EmpaToM is a video task assessing core social cognitive and affective functions including compassion and ToM. Neural activations and behavioral indices for these behavioral markers have been extensively validated using standard imaging paradigms and external behavioral measures including other published compassion and theory of mind paradigms (Kanske et al., 2016, Kanske et al., 2015).

During each trial of the EmpaToM, participants responded to a sequence of stimuli (see Fig. 2b). After a fixation cross (1–3 s), the name of a person (1 s) who would subsequently be speaking in a short video (∼15 s) was presented. The videos recounted autobiographic episodes, and differed in emotionality (emotionally neutral vs. negative contents) and in what question they gave rise to (ToM vs. nonToM). The emotional videos contained real-life stories in which the protagonists had suffered a lot and thus could induce compassion in the viewer. After each video, participants were asked to rate the valence of their own affective experience (on a scale from negative to positive; 4 s) and how much compassion they felt for the person in the previous video (scale from none to very much; 4 s). To assess theory of mind performance, after a fixation cross (1–3 s), a multiple choice question presented three response options, one of them being the correct answer as determined during stimulus development and piloting. The questions demanded either inference of mental state (i.e., thoughts, intentions, and beliefs) of the person in the video, or factual reasoning on the contents of the video. Participants had a maximum of 14 s to select one of the response options, which was then highlighted and remained on the screen for another second. After a fixation cross (0–2 s), a confidence rating was presented asking participants how confident they were when choosing the response in the previous question (4 s). Twelve trials per condition were presented, and participants were familiarized with the task in a short training session (4 trials).

Compassion was measured by means of compassion ratings across and within the neutral and emotional video conditions (see below). Since the conceptual understanding of compassion might change due to contemplative training (e.g., becoming aware of the difference between empathy and compassion), we ensured a consistent understanding by defining “compassion” during the EmpaToM training session as experiencing feelings of care, warmth, and benevolence towards another. ToM performance was measured by assessing performance (accuracy and RT) in the ToM questions (see below).

### 2.5 Statistical analysis

Data was analyzed using R software (R Core Team, 2013). For each measure, we calculated mean scores per participant and time point (descriptive statistics are provided in the Supplemental Material, Tables S3–S5). These scores were divided by the overall standard deviation to achieve comparability across measures. Change scores for each module and participant were then calculated by subtracting individual scores before each module from the scores at the end of each module (Tables S6–S8). To test the above hypotheses, these change scores were entered into linear mixed model analysis (see blow). This approach, relying on mixed effects modeling of change scores, was chosen because it avoids biasing module change estimates by including different participants before and after a module, while allowing inclusion of participants who did not provide datasets at all time points. Furthermore, change scores can be modeled directly as a function of the different modules (or retest) and these can be contrasted against each other. Linear mixed models are robust to unbalanced and incomplete data in longitudinal designs and account for potential within subject correlation induced by repeated measurements through the inclusion of random effects.

Scores in the cued flanker task were calculated as follows: Trials without a response within 200 to 1700 ms following target onset were discarded (4% of the trials). Mean error rates and reaction times of correct trials were calculated for the four task conditions (validly cued congruent targets, invalidly cued congruent targets, validly cued incongruent targets, invalidly cued incongruent targets). To assess general attentional capacities contributing to both executive control and reorienting, we calculated a difference score by subtracting the condition demanding both functions (invalidly cued incongruent targets) from the baseline condition (validly cued congruent targets). Thus, this score reflects to which extent accuracy (for error rates) or speed (for RT) is impaired by high attention demands, with higher scores indicating better performance. As a primary outcome measure, we computed an unweighted composite score of accuracy and RT by standardizing and averaging both measures (cf. Vandierendonck, 2017). Additionally, we tested for changes in both measures separately, accounting for multiple testing through Bonferroni correction. Furthermore, we also computed reorienting scores (validly cued congruent targets minus invalidly cued congruent targets) and executive control scores (validly cued congruent minus validly cued incongruent targets) that did not involve the interaction condition (invalidly cued incongruent targets) for isolated assessment of both functions (Trautwein et al., 2016). Descriptive statistics for these measures are provided in Table S3.

Scores for compassion were calculated by averaging across all (neutral and emotional) videos. This was based on the aim of loving-kindness meditation (a core practice of the Affect Module) to develop unconditional love, care, and kindness towards others, a quality that is paralleled by the feeling of compassion when confronted with suffering (Salzberg, 1995). Because the definition of compassion given to participants focused on the general positively valenced feeling of warmth, kindness, and concern (see above) and did not explicitly constrain it to situations of suffering, we expected a general increase in compassion across neutral and emotional videos. This expectation is in line with previous empirical findings showing an increase of positive feelings across high and low emotional stimuli conditions (Klimecki et al., 2013, Klimecki et al., 2014). We also explored whether increases would be driven by the emotional or neutral conditions, accounting for multiple testing through Bonferroni correction. Descriptive statistics for these measures are provided in Table S4.

Scores for ToM were calculated as follows: Trials without a response within the 14-second response window (3% of the trials) were treated as errors, since the inability to provide an answer to the ToM question with increasing time indicates poorer ToM ability. Error rates and reaction times for correct trials were averaged across all ToM questions to assess ToM performance, and across nonToM questions for a control score. As a primary outcome measure, we computed an unweighted composite score of error rate and RT by standardizing and averaging both measures (cf. Vandierendonck, 2017), in line with previous cross-sectional studies using the EmpaToM task (Kanske et al., 2016, Kanske et al., 2015). Additionally, we tested for changes in both measures separately, accounting for multiple testing by means of Bonferroni correction. Furthermore, we also assessed changes in factual reasoning questions to ensure that improvements would not be driven by general cognitive or motivational effects. Descriptive statistics for these measures are provided in Table S5.

First we assessed differential effects of the 3 modules separately for the three respective outcome measures and evaluated our hypotheses about specific effects of the three training modules on the targeted measures. Specifically, for attention we tested whether Presence effects were larger compared to retest and Affect effects. For compassion, we tested whether Affect effects were larger compared to retest, Presence and Perspective. Finally, for ToM we tested whether Perspective effects were larger compared to retest and Affect. Each module was always contrasted against effects of other modules and of retest only at the respective same time intervals. To this end, linear mixed models were estimated using the lme4 package (Bates, Mächler, Bolker, & Walker, 2015). Models included fixed effects for each time interval and module combination and random intercepts for participants. Specifically, for each outcome measure we fitted the following model to the change scores *Ci*:

$$C_{i}=\beta_{0}+\beta_{1}*retest_{2}+\beta_{2}*retest_{3}+\beta_{3}*Presence+\beta_{4}*Affect_{1}+$$

$$\beta_{5}*Affect_{2}+\beta_{6}*Affect_{3}+\beta_{7}*Perspective_{2}+\beta_{8}*Perspective_{3}$$

Note that the first retest interval (i.e., *retest1*) constitutes the intercept and all other effects are estimated in relation to this baseline. The fitted models then allowed us to test the above specified hypotheses, by contrasting the respective parameter estimates against each other (see Table S9 for a numeric specification of the contrasts).

Secondly, in order to test whether training module effects depended on time interval, we refitted the model with fixed effect factors for module (4 levels: retest, Presence, Affect, Perspective) and interval (3 levels: T0 to T1, T1 to T2, T2 to T3). Note that this model is equivalent to the first model in that it has the same amount of model parameters and the same model fit. Dependency of module effects on time interval (i.e., the interaction of module and interval) was then evaluated by comparing the full model against a model without the interaction term by means of chi-square likelihood ratio tests. A significant interaction would indicate that effects of Affect or Perspective might depend on the order in which they were completed (as first, second, or third module). In case of an interaction, we also report differential effects of the modules at each individual time interval.

As an estimate of effect size, we provide the model estimates (*b*) for each reported comparison. These are standardized effect estimates, since all dependent measures were divided by the overall standard deviation prior to model estimation. In addition, we report a supplementary table with retest controlled training effects following a procedure suggested by Morris (2008) for meta-analytical integration of results from pretest-posttest-control group designs. Specifically, mean change in the retest participants was subtracted from mean change in the training participants and divided by the pooled pretest standard deviation. Effects were classified according to standard conventions (i.e., small ≥ 0.20, medium ≥ 0.50, large ≥ 0.80).

Finally, we also assessed the effect of each module separately across the three outcome domains as another test of specificity of the training modules. Specifically, for each of the modules we tested to which extent it selectively influenced the targeted measure (i.e., attention, compassion, or ToM) and not the other measures, focusing on the markers that were most sensitive to the training effects. To this end, three separate mixed models were estimated for Presence, Affect, and Perspective Modules. The change scores pertaining to a given training module as well as retest scores from the same time intervals were entered into the model. Note that change scores were calculated from mean scores that had been divided by their standard deviation (see above), so that variance within each measure was equal. Models had fixed effects for intervention (one of the modules vs. retest) and outcome (attention, compassion, ToM) and random intercepts for participants. As retest effects might differ between the measures, the analysis focused on module by measure interactions, which would indicate that a module’s effects contrasted against retest effects differed between the measures.

Note that in the present analyses, estimates of the random intercepts were either zero or practically zero (≤1.759e^−15^), indicating that among subject variation was not present, likely due to the use of change scores. This allowed us to replicate the main results in classical linear models (Table S10).

Throughout the manuscript, all *p*-values are based on two-tailed statistical tests.

## 3 Results

First, we tested whether the different training modules have effects on the respective targeted outcome measures (Presence on attention, Affect on compassion, and Perspective on ToM) over and above effects of retest and the other training modules.

### 3.1 Training effects on attention

First we assessed training module effects on a general index of attentional capacity subsuming executive control and reorienting of attention using a composite of error rate and RT (see Fig. S1 A and Table S6). Effects of the Presence Module did not differ significantly from retest (*b* = 0.152, *z* = 1.314, *p* = .189) nor from the respective time interval (T0 to T1) for Affect (*b* = 0.196, *z* = 1.605, *p* = .108). Furthermore, module effects did not depend on the time interval, *X*^2^(3) = 3.639, *p* = .303, and none of the other comparisons was significant (p > .57). To detect effects that might be blurred by non-congruent effects on RT and error rate (Vandierendonck, 2017), we then tested both measures individually, applying Bonferroni correction for multiple testing. For error rates (Fig. 2f), improvements after the Presence Module differed from retest (*b* = 0.437, *z* = 3.856, *p_corr_* < .001) but no specific effect was found when comparing Presence and Affect at T0 to T1 (*b* = 0.035, *z* = 0.292, *p_corr_* = 1). A significant interaction between *interval* and *module*, *X*^2^(3) = 12.064, *p_corr_* = .014, indicated that module effects depended on the time interval and additional comparisons for individual intervals revealed that Affect also differed from retest at T0 to T1 (*b* = 0.402, *z* = 3.015, *p_corr_* = .005), but none of the comparisons after T1 were significant (all *p_corr_* > .28). Thus, the interaction reflects that Affect had a significant effect on error rate when applied as a first training module, whereas no further improvements were induced when applied after the Presence Module. The effect sizes for the observed reliable improvements in error rates after the three-month Presence Module from T0 to T1 were small in size (see Table S11). Regarding RT (Fig. S1 C, Table S6), Presence did not differ from retest (*b* = −0.185, *z* = −1.317, *p_corr_* = .375) nor from Affect (*b* = 0.29, *z* = 1.955, *p_corr_* = .101). Indicated by a significant interaction of *time* and *module*, *X*^2^(3) = 8.236, *p_corr_* = .041, we also tested other modules at individual time intervals, revealing a significant negative effect of Affect against retest at T0 to T1 (*b* = −0.475, *z* = −2.88, *p_corr_* = .008), but no significant effects at later time intervals (all *p_corr_* > .46).

Thus, compared to retest, the Affect Module had a positive effect on accuracy, but a negative effect on RT. In contrast, the Presence Module, when compared to retest, had a significant effect on accuracy, whereas it showed no relative loss in RT. As this pattern suggests that a shift in speed-accuracy trade-off might partially explain the results, we added changes in RT as an additional fixed effect to the error rate model. The RT covariate had a significant influence, *X^2^*(1) = 9.685, *p* < .002. The effect estimate indicated that improvements in error rate and RT scores correlated positively (*b* = 0.106), which argues against a general trade-off. Moreover, effects of Presence (*b* = 0.422, *z* = 3.821, *p* < .001) and Affect (*b* = 0.404, *z* = 3.109, *p =* .002) at T0 to T1 remained significant, and again no significant differences between Affect, Perspective, and retest were found at later intervals (all *p* > .26).

Based on a previous study demonstrating common attentional resources for reorienting and executive control (Trautwein et al., 2016), the previous analyses focused on a score that indexes performance under concurrent demand on these two functions. For completeness and to specifically investigate which of these attentional mechanisms profited most from the mental training, we also performed analyses separately for scores that distinguish executive control and stimulus-driven reorienting of attention (Table S6, Fig. S2 and Fig. S3). For executive control of attention, the analysis of composite scores showed a significant effect of Presence against retest (*b* = 0.31, *z* = 2.304, *p* = .021) but not against Affect (*b* = 0.012, *z* = 0.084, *p* = .933). Affect differed from retest at T0 to T1 (*b* = 0.330, *z* = 2.033, *p* = .042), though the interaction of *time* and *module* was not significant, *X^2^*(3) = 4.364, *p* = .225. Overall contrasts comparing Affect, Perspective and retest against each other were not significant (all *p* > .48). The Presence effects versus retest were driven by improvements in error rates (*b* = 0.435, *z* = 2.903, *p_corr_* = .007), not differing from Affect (*b* = 0.025, *z* = 0.158, *p_corr_* = 1), but Affect differing from retest at T0 to T1 (*b* = 0.41, *z* = 2.325, *p_corr_* = .04). The *module* by *time* interaction had no significant effect, *X^2^*(3) = 6.929, *p_corr_* = .148, and no significant overall differences were observed between Affect, Perspective and retest (all *p_corr_* > .784). Analyses of RT scores for executive control showed no training related effects (all *p_corr_* > .79) and there was no module by interval interaction, *X^2^*(3) = 1.676, *p_corr_* = 1.

Regarding reorienting of attention, composite scores showed no significant training effects (all *p* > .9) and there was no module by interval interaction, *X^2^* (3) = 1.295, *p* = .73. Analysis of individual error rate and RT scores did neither show significant interaction (both *p_corr_* < .66) nor significant training effects (all *p_corr_* > .062).

In sum, improvements in an index of general attentional performance seemed to occur after the Presence training, which were driven by improvements in executive control of attention, and reflected in error rates but not in RT scores. The first three months of Affect training were also associated with improvements in error rates, though accompanied by relative decreases in RT scores, potentially reflecting a change in speed-accuracy trade-off.

### 3.2 Training effects on compassion

To assess training effects on compassion, we analyzed trial-wise ratings of compassion in the EmpaToM task. As a primary outcome, we focused on compassion ratings averaged across trials. Overall contrasts of module effect estimates (Fig. 2g) across respective time intervals showed larger effects for Affect as compared to retest (*b* = 1.027, *z* = 4.420, *p* < .001), Perspective (*b* = 0.389, *z* = 1.973, *p* = .049) and Presence (*b* = 0.295, *z* = 2.542, *p* = .011). Furthermore, there was a significant interaction of *interval* and *module*, *X^2^*(3) = 9.604, *p* = .022, indicating that effects depended on the order of the training modules. Descriptively (Fig. 2d), compassion showed stronger increases after each Affect compared to the respective Perspective and retest intervals, however, the effect of Affect was most pronounced from T1 to T2 for TC1, the cohort that completed Affect before Perspective. Comparing effects between groups at individual time intervals revealed that, at T1 to T2, differences between Affect (TC1) and retest were significant (*b* = 0.677, *z* = 4.992, *p* < .001), but did not reach significance at T0 to T1 for TC3 (*b* = 0.129, *z* = 0.985, *p* = .325) or T2 to T3 for TC2 (*b* = 0.22, *z* = 1.626, *p* = 0.104). Similarly, at T1 to T2, differences between Affect (TC1) and Perspective (TC2) were significant (*b* = 0.319, *z* = 2.286, *p* = .022), but not at T2 to T3 when both cohorts had switched modules (*b* = 0.070, *z* = 0.503, *p* = .615). Finally, Perspective also differed from retest overall (*b* = 0.508, *z* = 2.631, *p* = .009) and at T1 to T2 in TC2 (*b* = 0.359, *z* = 2.610, *p* = .009), but not at T2 to T3 in TC1 (*b* = 0.150, *z* = 1.103, *p* = .27).

Effect sizes for the Affect Module increase in compassion were negligible at T0 to T1 (i.e., for the first three-month training in TC3), but large at T1 to T2 (for TC1 doing the Affect Module after the Presence Module), and small at T2 to T3 (for TC2 doing Affect after Presence and Perspective), while Perspective had a medium effect at T1 to T2 (in TC2) and a negligible effect at T2 to T3 (in TC1) (see Table S11).

While the previous analyses averaged across neutral and emotional videos, we ran additional follow-up analyses separately for ratings from neutral and emotional conditions (Fig. S4, Table S7), controlling for multiple comparisons. In the neutral video condition, results mirrored those from the averaged analysis, with significant differences between Affect and retest (*b* = 0.9698, *z* = 4.333, *p_corr_* < .001), Affect and Perspective (*b* = 0.5348, *z* = 2.816, *p_corr_* < .01), and Affect and Presence (*b* = 0.276, *z* = 2.462, *p_corr_* = .028). Again, there was a significant interaction, *X^2^*(3) = 13.258, *p_corr_* > .008, indicating that effects of Affect were most pronounced at T1 to T2. For this time interval, effects of Affect (TC1) against retest and Perspective (TC2) were significant at T1 to T2 (*p_corr_* < .007), but not at other time intervals (*p_corr_* > .41). Overall, the effect of Perspective against retest was not significant (*b* = 0.336, *z* = 1.807, *p_corr_* = .142); for individual comparisons it was significant at T1 to T2 (*b* = 0.311, *z* = 2.346, *p* = .038) in TC2, but not significant at T2 to T3 (*b* = .026, *z* = 0.197, *p_corr_* = 1). For emotional videos, Affect differed from retest (*b* = 0.836, *z* = 3.387, *p_corr_* = .001), while there was only a trend against Presence (*b* = 0.245, *z* = 1.984, *p_corr_* = .095), and no difference from Perspective (*b* = 0.101, *z* = 0.483, *p_corr_* = 1). Intervention effects did not depend on the time interval, *X^2^*(3) = 2.967, *p_corr_* = .793, and Perspective also differed from retest (*b* = 0.599, *z* = 2.918, *p_corr_* = .007).

Empathy is a construct that is closely related to, yet differentiable from compassion (Singer and Klimecki, 2014, Klimecki et al., 2014), and both concepts are assessed separately in the EmpaToM task (Kanske et al., 2015). We also tested whether the effects of the Affect Module would be specific to compassion (i.e., a feeling of warmhearted concern), or generalize to empathy (resonance with the other’s negative affect, defined as rated valence of ones own affect in the emotional minus neutral condition). This analysis showed no amplifying effects for the Affect Module on empathy, evidencing that effects of the Affect Module were specific to compassion (see Supplementary Fig. S5).

In sum, results are consistent with the hypothesis that Affect has the strongest effect on compassion, beyond retest, Presence and Perspective. Interestingly, these effects were driven by increases in the neutral video condition. And they were most pronounced for TC1 (i.e., at T1 to T2), potentially indicating that the module’s effectiveness depended on the order of training modules. Because the Perspective Module also led to increases in compassion, these improvements in TC2 after Perspective may have limited the effect that subsequent Affect training could still have on our measure of compassion.

### 3.3 Training effects on ToM

To test for training related improvements in cognitive perspective taking, we evaluated performance in ToM questions of the EmpaToM task by combining error rates and RT in a composite measure (see Fig. S6, Table S8). Numerically, Perspective differed from retest and Affect, but this effect only reached significance against Affect (*b* = 0.50, *z* = 2.376, *p* = .018), and not against retest (*b* = 0.32, *z* = 1.545, *p* = .122). Additional comparisons yielded no significant differences between Presence, Affect, and retest (all *p* > .19), and module effects did not depend on measurement interval, *X^2^*(3) = 2.548, *p* = .467).

To detect effects that might be blurred by non-congruent error rate and RT effects, we also assessed both measures in separation (Vandierendonck, 2017), correcting for multiple testing. For error rates, overall contrasts of module effects (Fig. 2h) showed a trend for Perspective compared to the respective retest intervals (*b* = 0.531, *z* = 2.032, *p_corr_* = .084) as well as compared to the Affect Module (*b* = 0.585, *z* = 2.196, p*_corr_* = .056). Effects did not depend on measurement interval, *X^2^*(3) = 0.219, *p_corr_* = 1) and no significant differences were found for additional comparisons not involving the Perspective module (i.e., between Presence, Affect, and retest) (all *p_corr_* = 1). For RT (Fig. S6, Table S8), Perspective differed neither from Affect nor from retest (p_corr_ > .75). There was no interaction of module and interval, *X^2^*(3) = 7.066, *p_corr_* = .13, and no significant difference between Affect, Presence and retest (all *p_corr_* > .15). Thus the partially significant effects of the Perspective Module on the composite score of ToM performance (differing significantly against the active control condition of the Affect Module but only descriptively from retest) seemed to be driven by improvements in error rates. These effects did not survive correction for multiple testing, but consistent trends were seen for both comparisons - against retest and against Affect. The effect size of these (non-significant) improvements in ToM performance after the three-month Perspective Modules was small (see Table S11).

Finally, to rule out the possibility that ToM improvements could reflect changes in domain general processes (e.g. memory, reasoning) also required by the task but not specific to ToM, we ran the same analyses on composite, reaction time and error rate scores from the nonToM factual reasoning control condition of the paradigm (Table S8). Perspective did not have a significant effect on any of these measures (composite: all *p* > .48; errors and RT all *p_corr_* > .23), suggesting that the results of an increase in ToM performance were specific to the ability to understand beliefs and intentions of other people rather than reflecting an increase in general cognitive capacities.

In sum, training-related improvements in ToM performance seemed to be specifically induced by the Perspective Module. This effect only reached statistical significance against the active control condition (Affect Module) and trends were observed against the control group. Note that, descriptively, all groups improved between T0 and T1 (see Fig. 2e), however, there were no significant differences between retest and any of the training groups suggesting that these changes are retest effects, that is, improvements due to performing the task the second time.

### 3.4 Differential effects of each module across three outcome measures

**Fig. 3:**
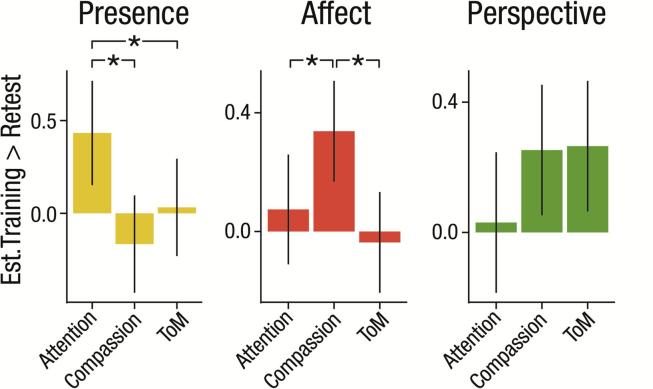
**Differential effects of each module across three outcome measures.** Contrasts of training vs. retest effects estimated from standardized change scores of attention, compassion and ToM. Estimates from three different models are shown assessing effects of Presence (T0 to T1 scores), Affect (T0 to T1, T1 to T2, and T2 to T3 scores), and Perspective (T1 to T2 and T2 to T3 scores). Error bars indicate 95% confidence intervals.

As another test of the specificity of the three different training modules, we compared the respective effects of a given module across all three outcome measures (Fig. 3), hypothesizing that the largest effects would be found within the outcome measure that was the target of a given module, that is, we expected strongest effects of Presence for attention, of Affect for compassion, and of Perspective for ToM measures. These analyses focused on error rate scores from the cued flanker and EmpaToM tasks, as the previous analyses demonstrated that improvements were specifically observed in accuracy and not in RT. Importantly, we focused on changes contrasted against retest, because change might differ between measures not only due to specific module effects but also due to susceptibility to retest effects. Separate mixed models were estimated for each training module (Presence, Affect, Perspective) with fixed effects for *intervention* (one of the modules vs. retest) and *outcome* (attention, compassion, ToM).

For Presence, a significant interaction of *intervention* and *outcome*, *X^2^*(2) = 9.747, *p* = .008, indicated that the module’s effects depended on the outcome. Linear contrasts revealed that training-related changes for Presence vs. retest were larger for attention as compared to compassion (*b* = 0.603, *z* = 3.078, *p* = .002) and ToM (*b* = 0.405, *z* = 2.068, *p* = .039). Presence vs. retest differences did not differ for compassion and ToM (*b* = −0.198, *z* = −1.045, *p* = .296). Thus, the Presence Module seems to be most efficient in increasing attention as compared to its effects on the other two dependent measures, compassion or ToM.

For Affect, a significant interaction of *intervention* and *outcome*, *X^2^*(2) = 9.816, *p* = .007, indicated dependency of the module’s effects on the outcome. Linear contrasts revealed that differences in training-related changes of the Affect Module vs. retest were larger for compassion as compared to attention (*b* = 0.264, *z* = 2.058, *p* = .04) and ToM (*b* = 0.375, *z* = 3.057, *p* = .002), while there was no difference between attention and ToM (*b* = 0.111, *z* = 0.866, *p* = .387). Again, these findings are in line with our hypothesis that the Affect Module should be most efficient in boosting compassion as compared to attention or ToM.

Descriptively, the Perspective Module had the strongest effects on ToM performance and on compassion. However, there was no significant interaction between *intervention* and *outcome*, *X^2^*(2) = 3.031, *p* = .22, and none of the specific comparisons were significant (all *p* > .118). Thus, while Perspective was the only module that led to a significant increase in ToM performance, this effect was statistically not significantly larger than the module’s effect on the other outcome measures.

## 4 Discussion

The present results show that daily contemplative mental training performed over several months can indeed induce specific plasticity in cognitive and social functions: Whereas attention was boosted most effectively by the Presence Module, increases in compassion were most pronounced after the Affect Module, and there was a significant effect of the Perspective Module on ToM performance when compared to the Affect Module and consistent trends when compared to retest. Effect sizes for these changes ranged from small to large depending on the outcome and the sequence of a given practice type in the entire nine-month longitudinal ReSource study (Singer et al., 2016). Taken together, the results decompose the broadly used concepts of meditation and mindfulness by directly demonstrating that different types of contemplative practices, often subsumed within a single program (Jazaieri et al., 2013, Kabat-Zinn, 1990, Mascaro et al., 2013), have specific effects on the mental faculties of attention, compassion, and ToM.

For the Presence Module focusing on cultivation of present-moment attention and interocepetive awareness through practices such as Breathing Meditation and Body Scan—which is thus most similar to the well-known MBSR program (Kabat-Zinn, 1990)—results are in line with previous findings of improved attention after mindfulness-based interventions (Allen et al., 2012, Slagter et al., 2007, Tang et al., 2007). These effects were constrained to the accuracy score in the cued flanker task (cf. Fig. 2f) and not observed for RT or a composite of both (cf. Fig. S1). Thus, performance accuracy improved, while RT remained constant, ruling out speed-accuracy trade-off. This finding is in line with several previous studies (Jo et al., 2016, Leonard et al., 2013, van den Hurk et al., 2010). Other studies that only reported RT scores of executive attention observed meditation related improvements in some cases (Ainsworth et al., 2013, Tang et al., 2007), but not in others (Wittmann et al., 2014). The effects of the Presence Module were restricted to attention, as the Module neither increased compassion nor ToM, that is, social capacities were not affected by these types of attention focused mindfulness practices, speaking against cascade-like models of mindfulness and emphasizing the need to explicitly cultivate intersubjective, compassion-based and ethical qualities (Brown and Ryan, 2004, Grossman, 2008, Neff and Germer, 2013). Interestingly, accuracy in the cued flanker task was also augmented by the Affect Module—which was also the most efficient module in increasing compassion. This finding suggests that in addition to targeting socio-emotional and motivational processes, practices of the Affect Module might also modulate attention. And indeed, the requirement of Loving-kindness Meditation (Salzberg, 1995), a core practice in the Affect Module as well as in other compassion-based intervention programs (Jazaieri et al., 2013, Neff and Germer, 2013), is to maintain a stable focus on a mental image (e.g., of a close person) while generating motivational states of loving-kindness. Note, however, that the improvement in accuracy for the Affect Module was accompanied by a relative slowing in RT. Furthermore, Affect did not have an additional effect on attention when practiced after the three-month Presence Module. Thus future research is needed to explore the nature of these effects of the Affect Module.

These findings bear relevance for future research on the treatment of a range of psychiatric disorders with deficiencies in attention (Tang et al., 2015). For example, the practices of the Presence Module might be suitable for children and adults suffering from ADHD (Cairncross & Miller, 2016), while practices fostering positive affect and possibly enhancing attention at the same time—as those in the Affect Module—might support treatment of affective disorders that are characterized by both, emotion and attention regulation difficulties (Hofmann et al., 2011, Posner et al., 2002).

The finding that the Affect Module led to strongest improvements in compassion—assessed as experienced feelings of care, warmth and benevolence—extends previous studies on kindness-based meditation showing increased positive affect (Engen and Singer, 2015b, Klimecki et al., 2013, Klimecki et al., 2014, Kok and Fredrickson, 2010). Importantly and in contrast to previous studies, participants were not instructed explicitly to apply the learned skills when performing the EmpaToM task, suggesting that the present findings represent trait changes in the tendency to spontaneously experience compassion for others. The finding that present-moment and attention-based mindfulness practices as taught in the Presence Module alone did not increase compassion suggests that explicit cultivation of intersubjective qualities such as empathy, gratitude, loving-kindness, and prosocial motivation is advisable to foster compassion – in line with the hypothesis that compassion is rooted in a care and affiliative, other-related motivational system (Engen and Singer, 2015a, Goetz et al., 2010, Klimecki et al., 2013, Klimecki et al., 2014). However, training-related effects on compassion were smaller for the group practicing Affect without first learning how to stabilize the mind in the Presence Module (cf. Fig. 2g). Thus, attention training during the Presence Module might prepare participants for the practices of the Affect Module. Interestingly, although the Affect Module was most efficient in boosting compassion, the Perspective Module also had a small but significant effect on compassion when compared to retest. Thus, spontaneously shifting perspective from oneself to other persons and understanding their intentions, beliefs and needs—as cultivated in the Perspective Module—might constitute an additional “socio-cognitive route” to fostering compassion (as has been debated in the literature (Dahl et al., 2015, Dahl et al., 2016, Engen and Singer, 2015a)). This is in line with results demonstrating that socio-affective and socio-cognitive processes do both contribute to prosocial action (Tusche, Bockler, Kanske, Trautwein, & Singer, 2016). Evidence for different pathways to foster a prosocial orientation is of particular relevance for populations with specific deficits in the tendency to empathize spontaneously – a deficit that can in extreme cases even be the underlying basis of serious aggressive assault (Meffert et al., 2013, Winter et al., 2017).

The Perspective Module was the only module that showed some evidence for an enhancing effect on ToM performance. When assessing an overall score of accuracy and RT (cf. Fig. S6) the effect of increased ToM performance after the 3-month Perspective Module was significant against the active control condition (i.e., the Affect Module), but not against retest, thus only partially confirming the hypothesis. Additional analyses on RT and accuracy scores showed trends for ToM accuracy when comparing Perspective to the Affect Module as well as to the retest control group (cf. Fig. 2h). Thus, these results provide only inconsistent evidence for an effect of the Perspective Module on ToM performance. Since the size of effects was small (TC1: *d* = 0.31; TC2: *d* = 0.23), one possible explanation for lacking significance against retest is that our study, though relying on a relatively large sample size, was still underpowered to consistently detect significant effects. Furthermore, other analyses correlating individual differences in ToM improvements with change in other variables seem to underscore the validity of observed ToM changes (Böckler et al., 2017, Valk et al., 2017, Valk et al., 2017): Valk et al. observed correlations between individual differences in ToM change and brain structure changes in areas that are typically known to be relevant for Theory of Mind (Valk et al., 2017, Valk et al., 2017). Furthermore, the Perspective Module involved both meditation and dyadic exercises which might differ in their effectiveness to enhance ToM, as the “Perspective Dyad” was specifically designed to train perspective taking skills on self and others. The dyadic exercise asked participants to describe situations from their daily life from the perspective of different inner parts and the listener had to guess which inner part of the other was speaking (see Methods section for details). Interestingly, we found that individual differences in the number of inner parts that the participants identified throughout the Perspective training predicted individual differences in improvements in ToM (Böckler et al., 2017), pointing towards the effectiveness of the Perspective Dyad as a tool to improve ToM. Thus, future studies testing the effects of practicing only the Perspective Dyad for an extended period of time may be promising for boosting ToM performance.

Taken together, these results do provide some limited evidence that specific types of mental training might be effective for increasing performance in higher-order cognitive perspective taking in a healthy adult sample without any deficits in ToM. Nevertheless, future research will need to explore the robustness of these findings. This is especially relevant since evidence for the trainability of ToM is rare. While some studies on training interventions for populations suffering from ToM deficits found positive results (Begeer et al., 2011, Lecce et al., 2015), a study with healthy adults only found effects of imitation-inhibition training on visual perspective taking, but not on performance in a classical ToM task (Santiesteban et al., 2012). Regarding contemplative mental training, two previous studies assessing inference of others’ emotional states from the eyes—a capacity that is closely related but nevertheless dissociable from ToM (Oakley et al., 2016)—yielded inconsistent results (Mascaro et al., 2013, Melloni et al., 2013). In contrast, the present results rely on a task validated for the specific assessment of high-level ToM performance (Kanske et al., 2016, Kanske et al., 2015), providing potential evidence for the malleability of high-level cognitive perspective taking. ToM deficits are associated with a range of clinical conditions, including autism, schizophrenia, and some forms of dementia (Brüne & Brüne-Cohrs, 2006), and also occur in healthy aging (Reiter, Kanske, Eppinger, & Li, 2017), resulting in a high demand for effective interventions. Therefore, future research on the robustness of the specific effect of the Perspective Module shown here, as well as on possibilities to increase effectiveness (e.g. longer training durations or optimized protocols focusing on the dyadic exercise) could inform such developments.

One possible limitation of the present results is that participants were not blind to the interventions (i.e. modules), which is in general difficult to realize in meditation studies and might lead to demand effects (Davidson & Kaszniak, 2015). One countermeasure suggested in the literature (Davidson and Kaszniak, 2015, Tang et al., 2015) is the use of active control conditions, which is realized most rigorously in the current study through the implementation of structurally equivalent training modules. Nevertheless, instructions on the mental training practices necessarily provided some knowledge of the modules’ general aims, which might have induced demand effects. However, several points argue against simple demand effects: First, the markers of attention and ToM were performance measures arguably being less prone to demand effects compared to self-report measures. Moreover, training effects did not occur in the control conditions of these tasks (i.e. factual reasoning questions in the EmpaToM; the congruent baseline condition in the attention task), and participants did not possess such a differentiated understanding of the complex protocol as to know where (after which module, in which trial, of which task) improvements are to be expected. With respect to the increase in compassion as measured in trial-wise self-report ratings (note that compassion, defined as a feeling, per definition requires some assessment of a subjective experience), we observed a pattern that was contrary to what would be expected if results were driven by social desirability. Specifically, increases in compassion were larger in the neutral video condition compared to the condition displaying emotional distress.

Finally, future research will need to explore the exact mechanisms of the different exercises within the training modules and disentangle their relative contributions to the observed changes in the three outcome measures. For example, both intersubjective training modules (Affect and Perspective) did not only contain classical meditation practices done by oneself (Loving-kindness and Observing Thoughts Meditation), but also so-called contemplative dyads practiced for 10 min with another partner as daily core practices supported by a web platform (Kok & Singer, 2017). Thus, future studies should aim to isolate effects of the meditation and dyad based practices. Furthermore, because practical considerations impeded the inclusion of another active training cohort only receiving the Perspective Module, the results are not conclusive about whether previous attention and interoception focused training as implemented in the Presence Module is needed for potential ToM benefits to emerge.

In sum, the present results have two crucial implications: First, our findings indicate that extended mental training effectively improves capacities that are crucial not only for individual flourishing, but also societal functioning at large. While executive control and attention are key predictors for educational success (Checa & Rueda, 2011), compassion and ToM contribute to adaptive social functioning and communication, prosocial behavior, and economic decision making (Goetz et al., 2010, Hein et al., 2016, Morishima et al., 2012, Weng et al., 2015). Second, the results show that the type of practice matters. Mindfulness practices focused on present-moment awareness improve attention, but are not efficient in enhancing socio-affective and socio-cognitive skills. While the capacity to understand beliefs, desires, and needs of others, a crucial capacity in cross-cultural dialogues, might potentially be trainable through specific perspective taking training, socio-affective practices are best to foster a loving and compassionate attitude towards others. These findings are not only relevant for the increasing number of people applying meditation techniques in their daily lives, with more then nine million practitioners in the US alone (Cramer et al., 2016). Such differential mapping of mental training effects also has promising implications for the development of refined intervention programs in education, health, and labor settings as well as for clinical populations with deficits in the domains of attention, social affect, or social cognition.
