# Supplementary material for: Differential benefits of mental training types for attention, compassion, and theory of mind
Source: Cognition. 2020 Jan;194:104039. doi: 10.1016/j.cognition.2019.104039 (PMC6891878; doi:10.1016/j.cognition.2019.104039)
Supplement: Supplementary data 1 [file mmc1.pdf]

Supplemental Material:

Differential Benefits of Mental Training Types for Attention, Compassion, and Theory of Mind

Short title: Training Attention, Compassion, and Theory of Mind

Authors: Fynn-Mathis Trautwein[1], Philipp Kanske[1], Anne Böckler-Raettig[1,2], Tania Singer[1]*

Affiliations:

[1] Edmont J. Safra Brain Research Center, University of Haifa, Haifa, Israel

[2] Clinical Psychology and Behavioral Neuroscience, Technische Universität Dresden, Chemnitzer Straße 46, 01187 Dresden, Germany.

[3] Department of Psychology, Würzburg University, Röntgenring 11, 97070 Würzburg, Germany.

[4] Max Planck Society, Social Neuroscience Lab, Berlin, Germany

*Correspondence to: ftrautwe@campus.haifa.ac.il.

Table S1. Dropout, missing data, and behavioral exclusion.

		TC1				TC2				TC3		RCC			
		T0	T1	T2	T3	T0	T1	T2	T3	T0	T1	T0	T1	T2	T3
Study	Dropout[a]	0	2	4	8	0	2	5	6	0	3	2	5	7	9
	Incomplete[b]	1	1	0	0	0	3	0	0	1	2	1	1	1	1
	MRI dropout[c]	0	2	7	6	0	2	4	4	0	3	1	6	9	7
Empa-ToM	Missing[d]	3	6	2	4	4	7	6	4	6	4	5	4	1	2
	Final N	76	69	67	62	77	67	66	67	74	69	81	74	72	71
Attention Task	Missing[d]	3	2	4	4	4	5	5	2	8	3	4	1	1	1
	Excluded[e]	7	9	7	6	9	9	8	12	6	9	3	9	6	6
	Final N	69	64	58	56	68	60	59	57	66	61	79	68	66	66

[a] Cumulative dropout or exclusion due to medical reasons, discomfort with study or experiments, time constraints, or other reasons (see Singer et al., 2016, for details).

[b] EmpaToM and CueFla experiments not completed because participants dropped out during the measurement phase. Participants were classified as dropout for a timepoint if no data at all were present; participants were counted as incomplete if they left the study during a measurement phase before the MRI scanning session.

[c] Cumulative dropout from MRI measurements because of medical reasons or discomfort (see Singer et al., 2016). Participants who later dropped out of the entire study where not counted as MRI dropouts anymore.

[d] Missing data due to technical, scheduling, or health issues.

[e] Excluded due to incorrect or poor task performance (error-rate in one of the experimental blocks above 50%; percentage of misses above 12.5% (cf. Trautwein et al., 2016)).

Table S2. Sample description for available change scores.

		T0 to T1			T1 to T2			T2 to T3		
		RT	PRE	AFF	RT	AFF	PER	RT	AFF	PER
EmpaToM	N	73	133	68	68	64	61	70	63	62
	Age	40.0	40.8	39.6	39.6	40.4	40.3	40.0	40.7	40.6
	Female	39	77	38	36	34	36	38	37	31
Attention Task	N	65	113	55	60	53	53	62	49	51
	Age	40.3	40.9	40.1	39.6	41.3	40.5	40.4	39.9	41.3
	Female	35	61	33	32	27	30	32	27	26

Note: RT = Retest, PRE = Presence module, AFF = Affect module, PER = Perspective module.

Table S3. Descriptive statistics for performance in the cued flanker task.

		Timepoint							
		T0		T1		T2		T3	
	Group	mean	sd	mean	sd	mean	sd	mean	sd
General Attention Composite Score	TC1	-0,06	0,86	0,13	0,87	0,12	0,87	0,26	0,76
	TC2	-0,06	0,78	0,13	0,69	0,20	0,69	0,41	0,68
	TC3	-0,27	0,74	-0,13	0,93				
	RCC	-0,31	0,88	-0,11	0,86	-0,05	0,83	-0,05	0,83
Executive Attention Composite Score	TC1	-0,24	1,03	0,18	0,75	0,09	0,59	0,10	0,73
	TC2	-0,09	0,87	0,23	0,62	0,21	0,55	0,20	0,63
	TC3	-0,26	1,09	-0,03	1,01				
	RCC	-0,21	0,97	-0,05	0,76	0,00	0,81	0,03	0,72
Reorienting Composite Score	TC1	-0,05	0,84	0,15	0,68	0,09	0,69	0,10	0,63
	TC2	-0,15	0,83	0,02	0,58	0,11	0,57	0,25	0,57
	TC3	-0,22	0,94	-0,11	0,62				
	RCC	-0,13	0,82	-0,04	0,67	0,02	0,58	0,08	0,66
General Attention Accuracy Score	TC1	-0.26	0.21	-0.22	0.19	-0.23	0.20	-0.20	0.19
	TC2	-0.24	0.20	-0.18	0.13	-0.17	0.16	-0.16	0.16
	TC3	-0.32	0.21	-0.27	0.23				
	RCC	-0.28	0.22	-0.29	0.22	-0.28	0.21	-0.29	0.23
Executive Attention Accuracy Score	TC1	-0.05	0.07	-0.02	0.05	-0.03	0.04	-0.04	0.05
	TC2	-0.04	0.06	-0.02	0.03	-0.02	0.03	-0.03	0.04
	TC3	-0.05	0.07	-0.04	0.06				
	RCC	-0.05	0.06	-0.05	0.05	-0.05	0.05	-0.04	0.06
Reorienting Accuracy Score	TC1	-0.04	0.07	-0.03	0.05	-0.02	0.05	-0.03	0.06
	TC2	-0.04	0.06	-0.03	0.05	-0.02	0.05	-0.01	0.05
	TC3	-0.04	0.09	-0.04	0.07				
	RCC	-0.04	0.07	-0.03	0.06	-0.03	0.05	-0.03	0.05
General Attention Reaction Time Score	TC1	-214.88	87.68	-196.58	96.31	-196.20	82.03	-184.73	76.87
	TC2	-221.27	76.46	-216.23	82.84	-206.97	75.58	-177.09	74.10
	TC3	-223.29	85.87	-221.00	83.32				
	RCC	-249.00	97.82	-209.83	92.90	-204.19	77.39	-202.34	75.25
Executive Attention Reaction Time Score	TC1	-81.42	45.19	-65.78	37.82	-62.58	28.37	-63.01	35.10
	TC2	-76.38	43.30	-67.55	35.94	-68.62	31.69	-62.25	28.51
	TC3	-79.11	44.00	-69.41	37.91				
	RCC	-82.50	39.78	-65.97	35.51	-62.96	38.16	-63.39	32.17
Reorienting Reaction Time Score	TC1	-107.41	67.30	-104.80	65.93	-114.35	69.68	-103.09	55.70
	TC2	-124.14	65.90	-124.31	55.90	-115.90	51.19	-100.83	55.42
	TC3	-121.35	60.65	-123.53	54.13				
	RCC	-131.50	70.30	-117.23	62.95	-113.96	50.44	-110.94	53.52

Note: Values constitute difference scores between experimental conditions; see the methods section in the main text for details. Accuracy scores are the ratio of correct responses vs. all responses in a condition. Reaction time is specified in ms.

Table S4. Descriptive statistics for compassion ratings in the EmpaToM.

		Timepoint							
		T0		T1		T2		T3	
	Group	mean	sd	mean	sd	mean	sd	mean	sd
Compassion Rating Overall	TC1	3.47	0.59	3.29	1.05	3.75	0.84	3.84	0.85
	TC2	3.45	0.60	3.51	0.59	3.63	0.69	3.82	0.77
	TC3	3.36	0.84	3.50	0.83				
	RCC	3.37	0.89	3.39	0.76	3.28	0.82	3.25	0.94
Compassion Rating Neutral Videos	TC1	2.68	0.83	2.51	1.28	3.15	0.94	3.22	0.99
	TC2	2.71	0.85	2.82	0.78	2.95	0.94	3.21	0.91
	TC3	2.58	1.04	2.80	0.99				
	RCC	2.55	1.12	2.62	1.05	2.49	1.08	2.53	1.19
Compassion Rating Emotional Videos	TC1	4.27	0.58	4.07	1.00	4.35	0.86	4.45	0.81
	TC2	4.18	0.64	4.20	0.66	4.31	0.69	4.43	0.79
	TC3	4.15	0.94	4.21	0.82				
	RCC	4.18	0.87	4.17	0.71	4.07	0.82	3.96	0.88

Table S5. Descriptive statistics for performance in the EmpaToM.

		Timepoint							
		T0		T1		T2		T3	
	Group	mean	sd	mean	sd	mean	sd	mean	sd
ToM Questions Composite Score	TC1	-0,19	0,79	0,06	0,70	0,06	0,79	0,21	0,79
	TC2	-0,15	0,81	-0,08	0,79	0,09	0,73	0,14	0,73
	TC3	-0,07	0,75	0,13	0,77				
	RCC	-0,14	0,81	-0,06	0,64	0,02	0,76	0,08	0,74
ToM Questions Accuracy	TC1	0.63	0.13	0.68	0.12	0.68	0.12	0.7	0.13
	TC2	0.65	0.12	0.67	0.15	0.7	0.12	0.71	0.12
	TC3	0.68	0.11	0.71	0.12				
	RCC	0.65	0.13	0.69	0.11	0.69	0.13	0.7	0.13
ToM Questions Reaction Time	TC1	8.38	1.47	8.26	1.31	8.18	1.26	8.11	1.19
	TC2	8.45	1.29	8.46	1.06	8.31	1.24	8.33	1.13
	TC3	8.54	1.24	8.34	1.39				
	RCC	8.46	1.37	8.62	1.12	8.41	1.22	8.34	1.24
Control Questions Composite	TC1	-0,07	0,78	-0,08	0,80	0,21	0,76	0,22	0,76
	TC2	-0,19	0,80	-0,03	0,85	0,09	0,75	0,04	0,82
	TC3	-0,17	0,80	0,08	0,74				
	RCC	-0,13	0,72	-0,10	0,74	0,09	0,76	0,14	0,70
Control Questions Accuracy	TC1	0.56	0.15	0.57	0.17	0.63	0.17	0.63	0.15
	TC2	0.56	0.17	0.59	0.19	0.63	0.15	0.63	0.17
	TC3	0.57	0.15	0.61	0.14				
	RCC	0.56	0.16	0.6	0.15	0.62	0.15	0.62	0.16
Control Questions Reaction Time	TC1	8,33	1,38	8,41	1,11	8,18	1,17	8,14	1,17
	TC2	8,61	1,19	8,49	1,21	8,48	1,18	8,57	1,20
	TC3	8,66	1,27	8,35	1,26	NaN	NA	NaN	NA
	RCC	8,48	1,33	8,73	1,10	8,43	1,23	8,31	1,06

Note: Accuracy scores are the ratio of correct responses vs. all responses in a condition. Reaction time is specified in seconds.

Table S6. Descriptive statistics for change scores of performance in the cued flanker task.

		Time interval					
		T0 to T1		T1 to T2		T2 to T3	
	Group	mean	sd	mean	sd	mean	sd
General Attention Composite Score	TC1	0,31	0,69	0,01	0,57	0,21	0,69
	TC2	0,26	0,84	0,08	0,65	0,17	0,69
	TC3	0,09	0,86				
	RCC	0,14	0,85	0,23	0,78	0,05	0,70
Executive Attention Composite Score	TC1	0,46	1,04	-0,05	0,83	-0,02	0,80
	TC2	0,36	0,94	-0,02	0,85	0,00	0,68
	TC3	0,43	1,12				
	RCC	0,10	0,91	0,10	0,77	-0,01	0,82
Reorienting Composite Score	TC1	0,26	1,06	0,01	1,16	0,01	1,08
	TC2	0,30	1,06	0,16	0,91	0,13	0,92
	TC3	0,11	1,14				
	RCC	0,02	1,12	0,14	0,95	0,06	0,94
General Attention Accuracy Score	TC1	0,25	0,75	-0,05	0,70	0,19	0,74
	TC2	0,31	0,81	0,02	0,64	0,00	0,64
	TC3	0,24	0,81				
	RCC	-0,16	0,77	0,15	0,68	0,00	0,71
Executive Attention Accuracy Score	TC1	0,33	1,03	-0,11	0,87	-0,05	0,91
	TC2	0,29	1,05	0,03	0,75	-0,14	0,78
	TC3	0,29	1,13				
	RCC	-0,12	0,96	0,04	1,04	0,03	1,00
Reorienting Accuracy Score	TC1	0,25	1,33	0,24	1,23	-0,31	1,26
	TC2	0,24	0,98	0,11	0,99	0,10	0,97
	TC3	0,11	1,27				
	RCC	-0,03	1,18	0,03	1,10	-0,01	1,11
General Attention Reaction Time Score	TC1	0,27	0,80	0,07	0,76	0,15	0,80
	TC2	0,12	0,95	0,11	0,80	0,28	0,80
	TC3	-0,09	1,08				
	RCC	0,38	1,06	0,23	1,05	0,07	0,78
Executive Attention Reaction Time Score	TC1	0,43	1,11	0,03	0,76	0,01	0,94
	TC2	0,32	1,12	-0,06	0,99	0,14	0,65
	TC3	0,42	1,18				
	RCC	0,29	1,05	0,12	0,67	-0,05	0,77
Reorienting Reaction Time Score	TC1	0,12	0,88	-0,23	1,04	0,32	0,91
	TC2	0,19	1,28	0,12	1,04	0,09	0,77
	TC3	0,05					
	RCC	0,06	1,01	0,17	0,89	0,10	0,71

Note: Values are based on difference scores between experimental conditions; see the methods section in the main text for details. Change scores were calculated by dividing each score by the overall standard deviation and then subtracting pre from post values. Accuracy scores are based on the ratio of correct responses vs. all responses in a condition. Reaction time is specified ms.

Table S7. Descriptive statistics for change scores of EmpaToM compassion ratings.

		Time interval					
		T0 to T1		T1 to T2		T2 to T3	
	Group	mean	sd	mean	sd	mean	sd
Compassion Rating Overall	TC1	-0.23	0.94	0.53	0.91	0.13	0.65
	TC2	-0.01	0.68	0.21	0.70	0.20	0.69
	TC3	0.17	0.87				
	RCC	0.04	0.84	-0.15	0.67	-0.02	0.72
Compassion Rating Neutral Videos	TC1	-0.22	1.01	0.34	0.86	0.15	0.73
	TC2	-0.06	0.72	0.20	0.69	0.11	0.73
	TC3	0.11	0.92				
	RCC	-0.03	0.92	-0.13	0.72	-0.12	0.87
Compassion Rating Emotional Videos	TC1	-0.19	0.91	0.57	0.91	0.09	0.59
	TC2	0.02	0.69	0.17	0.72	0.23	0.65
	TC3	0.19	0.82				
	RCC	0.09	0.83	-0.14	0.66	0.06	0.63

Note: Change scores were calculated by dividing each score by the overall standard deviation and then subtracting pre from post values.

Table S8. Descriptive statistics for change scores of performance in the EmpaToM task.

		Time interval					
		T0 to T1		T1 to T2		T2 to T3	
	Group	mean	sd	mean	sd	mean	sd
ToM Questions Composite Score	TC1	0,32	0,91	-0,02	0,73	0,22	0,94
	TC2	0,14	0,83	0,25	0,78	-0,01	0,80
	TC3	0,25	0,83	NaN	NA	NaN	NA
	RCC	0,07	0,80	0,06	0,79	0,09	0,90
ToM Questions Accuracy	TC1	0,41	1,13	-0,07	0,98	0,27	0,98
	TC2	0,17	1,20	0,28	0,97	0,03	1,05
	TC3	0,21	1,05				
	RCC	0,26	0,96	-0,02	1,07	0,04	1,10
ToM Questions Reaction Time	TC1	-0,07	0,95	-0,04	0,78	-0,07	0,81
	TC2	-0,05	0,76	-0,10	0,90	0,05	0,83
	TC3	-0,18	0,79				
	RCC	0,15	0,88	-0,11	0,59	-0,10	0,74
Control Questions Composite Score	TC1	-0,03	0,84	0,33	0,92	0,03	0,87
	TC2	0,19	0,86	0,17	0,80	-0,11	0,83
	TC3	0,27	0,89				
	RCC	-0,02	0,75	0,26	0,80	0,09	0,74
Control Questions Accuracy	TC1	0,03	1,01	0,37	1,08	0,01	0,99
	TC2	0,16	1,03	0,27	0,97	-0,04	1,07
	TC3	0,18	0,92				
	RCC	0,18	1,00	0,19	0,96	-0,01	0,93
Control Questions Reaction Time	TC1	0,08	0,86	-0,14	0,80	-0,04	0,84
	TC2	-0,13	0,81	0,00	0,81	0,13	0,83
	TC3	-0,23	0,90				
	RCC	0,21	0,90	-0,21	0,73	-0,14	0,73

Note: Change scores were calculated by dividing each score by the overall standard deviation and then subtracting pre from post values.

Table S9. Contrast coding for hypothesis tests.

		Parameter Estimate								
		T0 to T1			T1 to T2			T2 to T3		
		RT	PRE	AFF	RT	AFF	PER	RT	AFF	PER
		β_0	β_3	β_4	β_1	β_5	β_7	B_2	β_6	β_8
Attention	PRE > RT	-1	1	0	0	0	0	0	0	0
	PRE > AFF	0	1	-1	0	0	0	0	0	0
Compassion	AFF > RT	-1	0	1	-1	1	0	1	-1	0
	AFF > PRE	0	-1	1	0	0	0	0	0	0
	AFF > PER	0	0	0	0	1	-1	0	1	-1
ToM	PER > RT	0	0	0	-1	0	1	-1	0	1
	PER > AFF	0	0	0	0	-1	1	0	-1	1

Note: RT = Retest, PRE = Presence module, AFF = Affect module, PER = Perspective module; β refers to the respective estimates defined by the mixed model formula [$C_i = \beta_0 + \beta_1 * retest_2 + \beta_2 * retest_3 + \beta_3 * Presence + \beta_4 * Affect_1 + \beta_5 * Affect_2 + \beta_6 * Affect_3 + \beta_7 * Perspective_2 + \beta_8 * Perspective_3$].

Table S10. Replication of main results in linear models.

		Attention (accuracy score)			Compassion (mean ratings)			ToM (accuracy score)		
Interval	Contrast	*b*	*t*	*p*-value (corr.)	*b*	*t*	*p*-value	*b*	*t*	*p*-value (corr.)
T0 → T1	PRE > RT	0.437	3.856	0.000	-0.166	-1.462	0.144	0.032	0.209	1.000
	AFF > RT	0.402	3.015	0.005	0.129	0.985	0.325	-0.055	-0.309	1.000
	PRE > AFF	0.035	0.292	1.000	-0.295	-2.542	0.011	0.087	0.554	1.000
T1 → T2	AFF > RT	-0.201	-1.462	0.289	0.677	4.992	0.000	-0.049	-0.267	1.000
	PER > RT	-0.127	-0.926	0.710	0.359	2.610	0.009	0.303	1.629	0.208
	AFF > PER	-0.074	-0.520	1.000	0.319	2.286	0.023	-0.352	-1.865	0.125
T2 → T3	AFF > RT	-0.003	-0.021	1.000	0.220	1.626	0.104	-0.005	-0.029	1.000
	PER > RT	0.189	1.373	0.341	0.150	1.103	0.270	0.228	1.243	0.429
	AFF > PER	-0.192	-1.317	0.377	0.070	0.503	0.615	-0.234	-1.240	0.431
T1 → T3	PER > RT	0.062	0.318	1.000	0.508	2.631	0.009	0.531	2.032	0.085
	AFF > PER	-0.266	-1.308	0.383	0.389	1.973	0.049	-0.585	-2.196	0.057
T0 → T3	AFF > RT	0.199	0.839	0.804	1.027	4.420	0.000	-0.109	-0.348	1.000

Table S11. Effect sizes for each module and time interval.

	Time interval					
	T0 to T1		T1 to T2		T2 to T3	
	Presence	Affect	Affect	Perspective	Affect	Perspective
Attention (accuracy)	0.43	0.39	-0.19	-0.13	0.00	0.18
Compassion (mean rating)	-0.19	0.12	0.60	0.42	0.24	0.15
ToM (accuracy)	0.03	-0.06	-0.05	0.31	-0.01	0.23

Figure S1. General Attention Score.

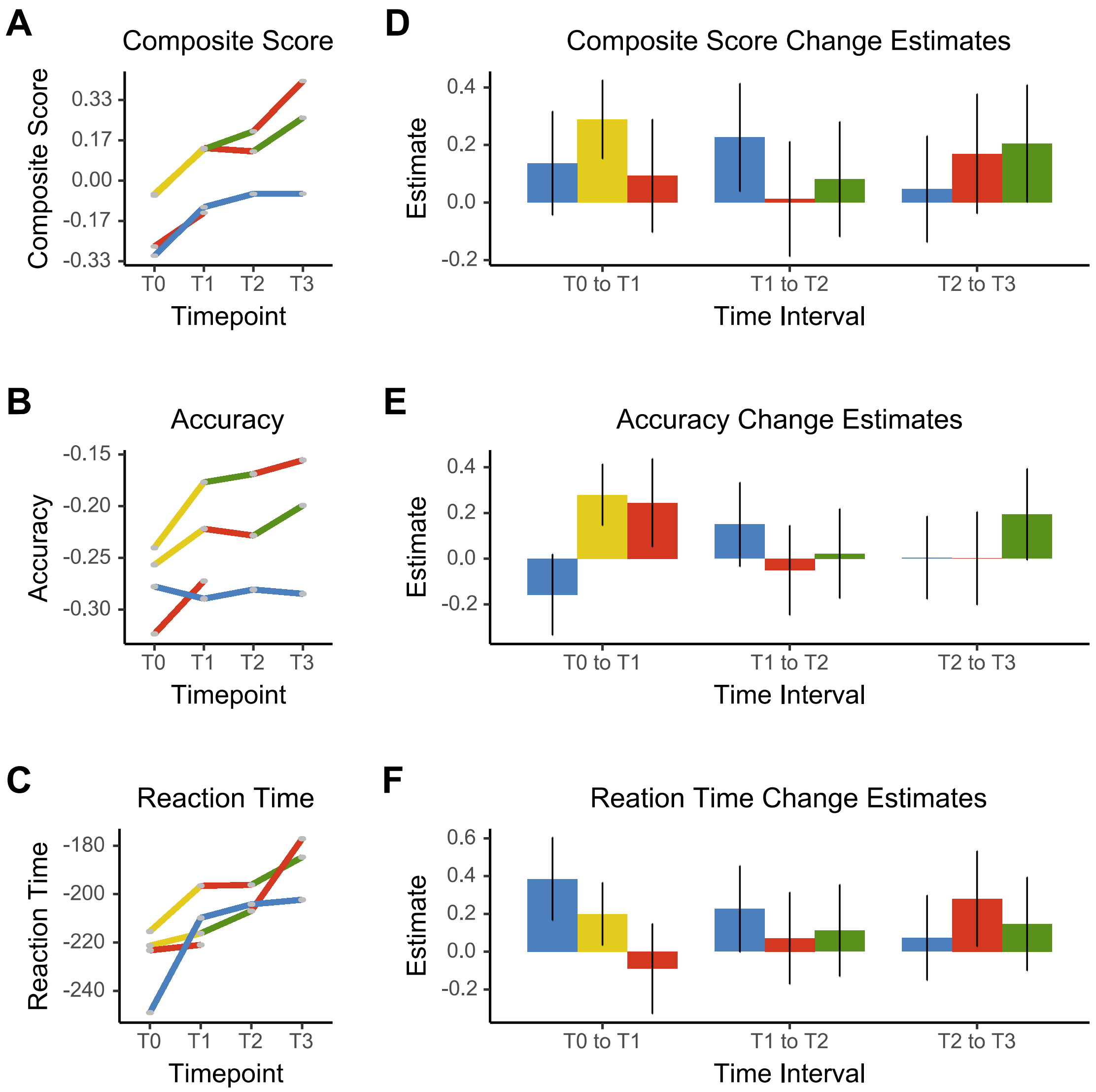

Fig. S1. Descriptive data and model estimates for the general attention score. Scores represent differences of performance in the reorienting and conflict condition minus baseline condition. Panels on the left show descriptive plots of mean values for (**a**) the composite of accuracy and reaction time, (**b**) raw accuracy scores and (**c**) reaction time. The mean values of each individual were used to calculate change scores for each available pair of two consecutive time points, which were used to estimate effects for retest and the three training modules shown in (**f-h**). Error bars indicate 95% confidence intervals. See the main text for details on statistical tests.

Figure S2. Executive Control of Attention.

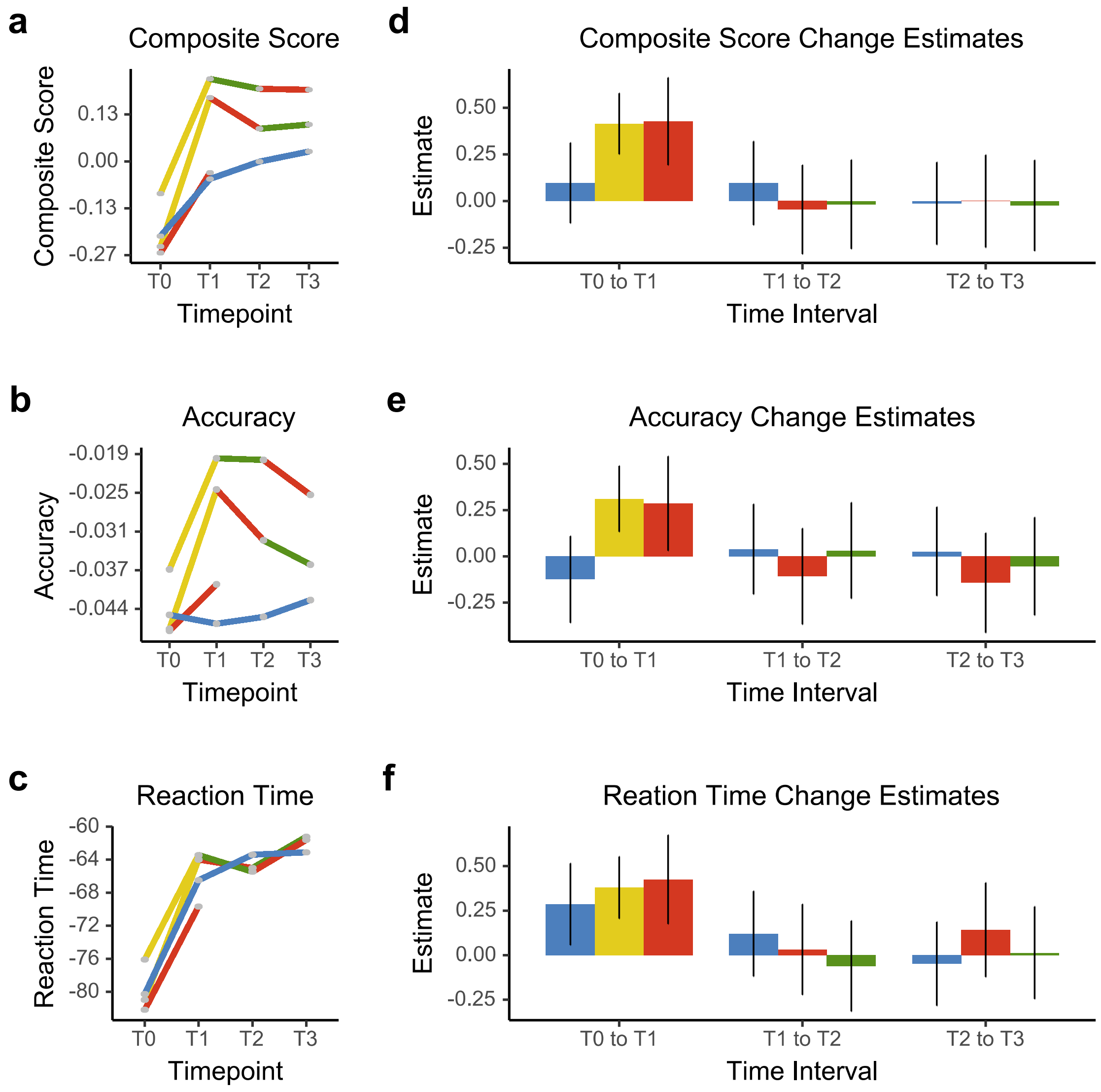

Fig. S2. Descriptive data and model estimates for executive control of attention. Scores represent differences of performance in the conflict condition minus baseline condition. Panels on the left show descriptive plots of mean values for (**a**) the composite of accuracy and reaction time, (**b**) raw accuracy scores and (**c**) reaction time. The mean values of each individual were used to calculate change scores for each available pair of two consecutive time points, which were used to estimate effects for retest and the three training modules shown in (**f-h**). Error bars indicate 95% confidence intervals. See the main text for details on statistical tests.

Figure S3. Reorienting of Attention.

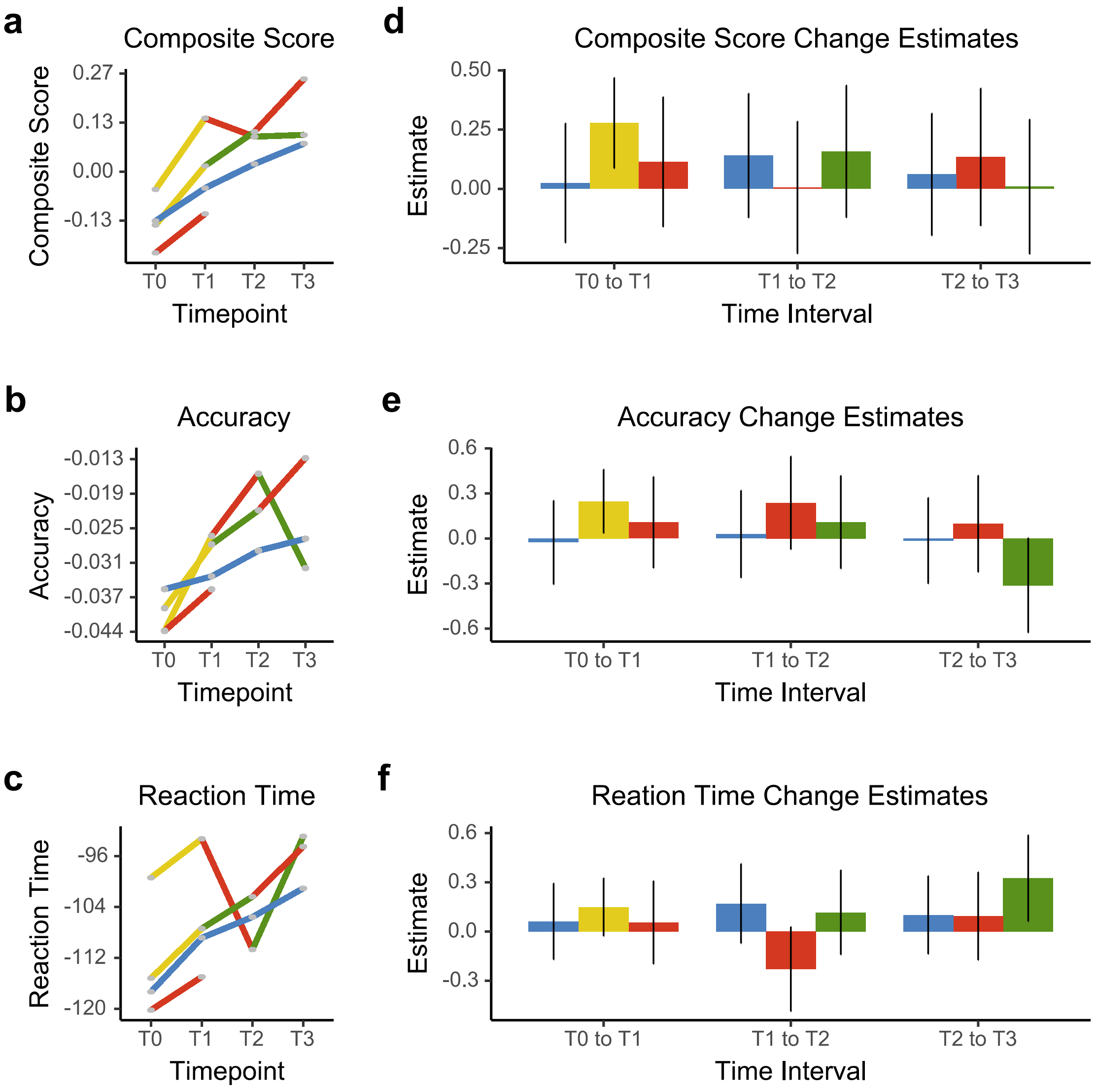

Fig. S3. Descriptive data and model estimates for reorienting of attention. Scores represent differences of performance in the reorienting condition minus baseline condition. Panels on the left show descriptive plots of mean values for (**a**) the composite of accuracy and reaction time, (**b**) raw accuracy scores and (**c**) reaction time. The mean values of each individual were used to calculate change scores for each available pair of two consecutive time points, which were used to estimate effects for retest and the three training modules shown in (**f-h**). Error bars indicate 95% confidence intervals. See the main text for details on statistical tests.

Figure S4. Compassion Ratings.

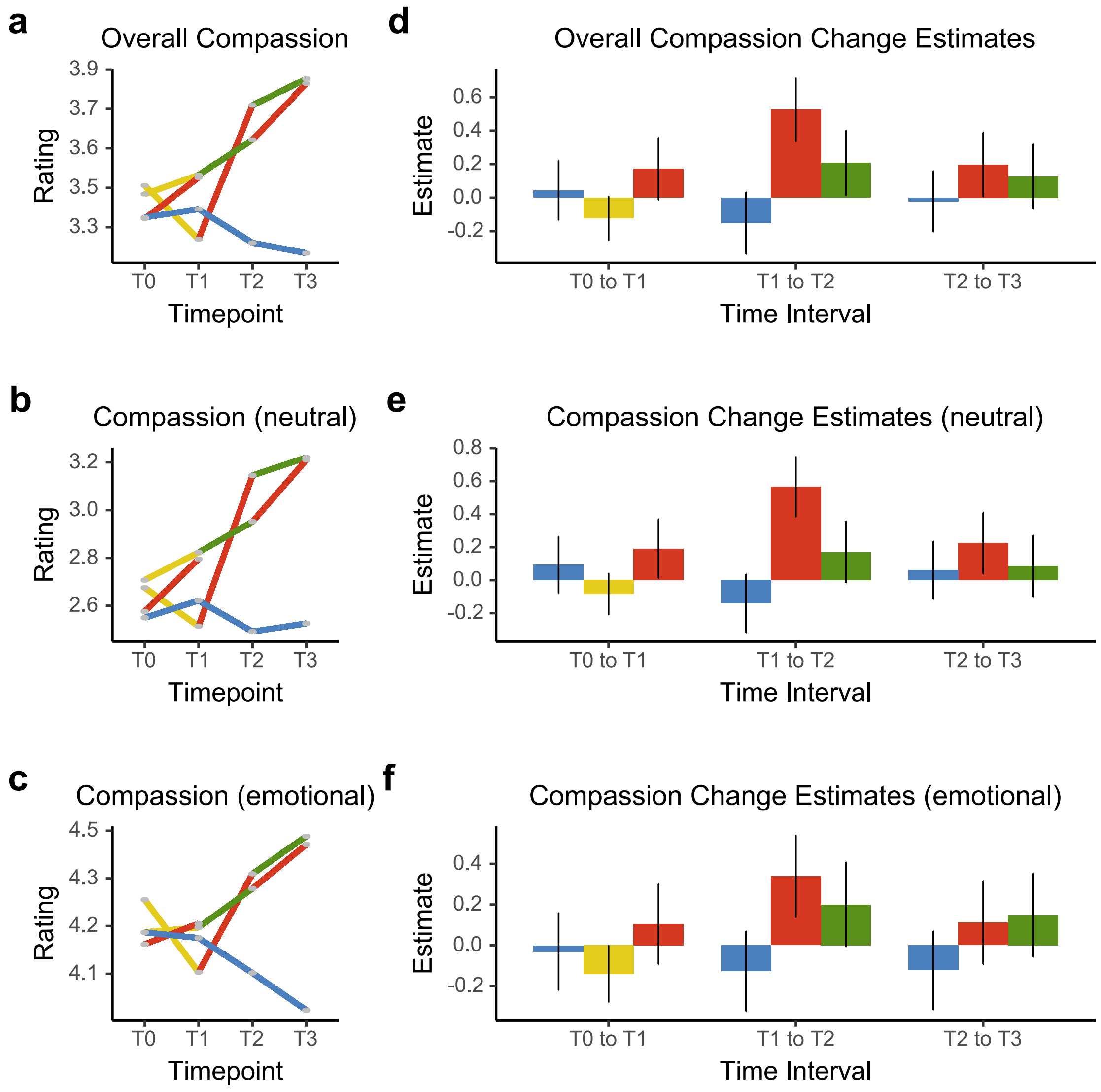

Fig. S4. Descriptive data and model estimates for compassion ratings. Scores represent ratings on a continuous visual scale (ranging from 0 to 6). Panels on the left show descriptive plots of mean values for (**a**) ratings across both neutral and emotional video conditions, (**b**) ratings after neutral videos (**c**) ratings after emotional videos. The mean values of each individual were used to calculate change scores for each available pair of two consecutive time points, which were used to estimate effects for retest and the three training modules shown in (**f-h**). Error bars indicate 95% confidence intervals. See the main text for details on statistical tests.

Figure S5. Valence ratings (empathy).

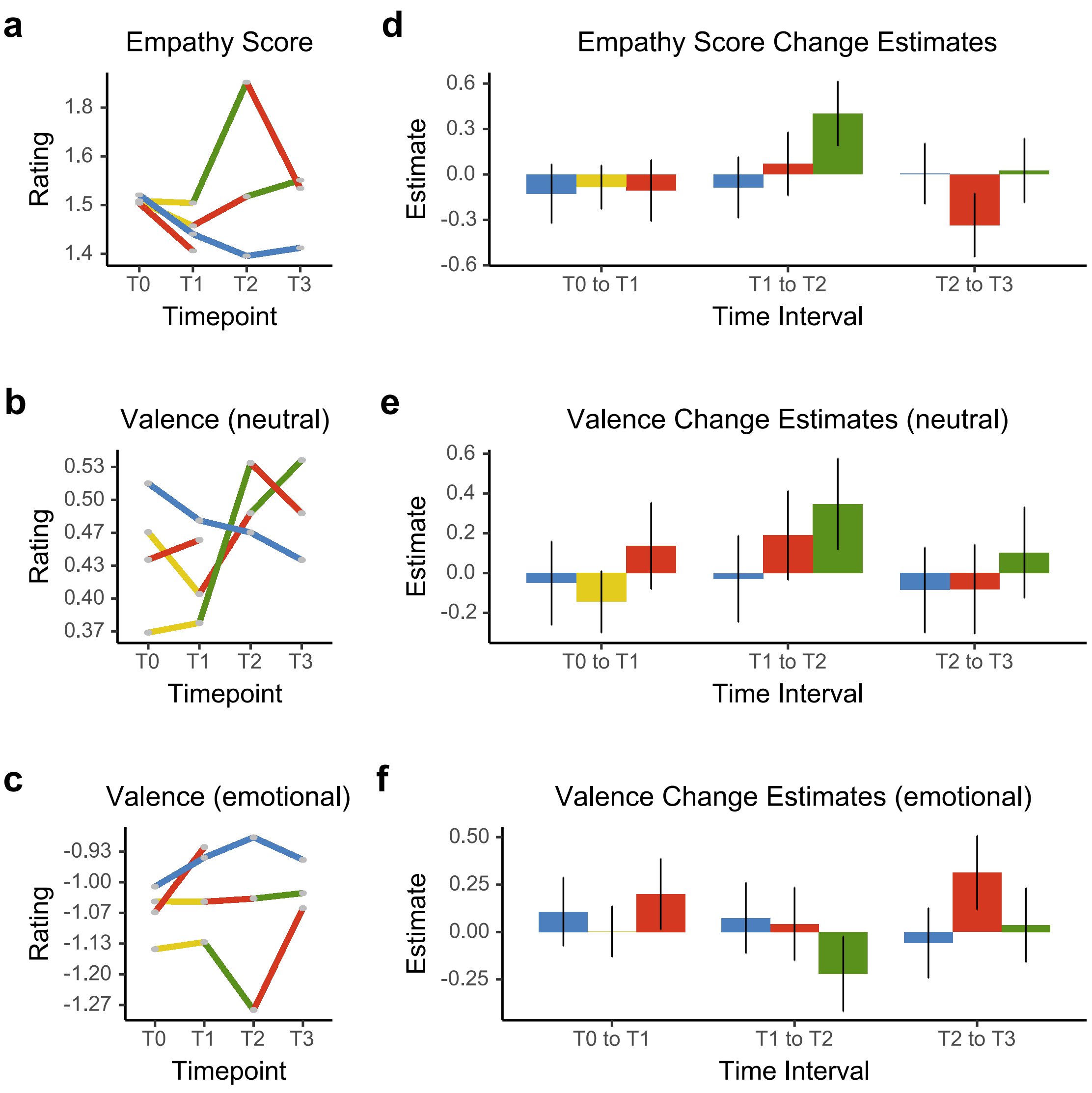

Fig. S5. Descriptive data and model estimates for valence rating. Scores represent ratings on a continuous visual scale of affective valence ranging from negative (-3) to positive (3). Panels on the left show descriptive plots of mean values for (**a**) empathy scores computed as the difference of ratings in the emotional minus neutral video condition, (**b**) reversed ratings after neutral videos (**c**) reversed ratings after emotional videos. The mean values of each individual were used to calculate change scores for each available pair of two consecutive time points, which were used to estimate effects for retest and the three training modules shown in (**f-h**). Error bars indicate 95% confidence intervals. Statistical analysis yielded no significant difference between Affect and Retest (b = -0.165, z = -0.649, p = .517), however, there was a significant difference between Perspective and Affect (b = -0.693, z = -3.217, p = .001). Furthermore, the interaction of module and time interval was marginally significant, $X^2(3) = 7.578$, p = .056). As shown in the figure, there was a steep increase after the Perspective Module when completed before Affect, and the effect was reversed by the following Affect Module. Tests of individual time intervals showed that this increase for Perspective (at T1 to T2) was significant compared to retest (b = 0.487, z = 3.241 , p = .001) and to Affect (b = 0.332,

$z = 2.181$, $p = .029$), as was the reversal for Affect (at T2 to T3) against retest ($b = -0.34$, $z = -2.301$, $p = .021$) and against Perspective ($b = -0.361$, $z = -2.369$, $p = .018$). Other contrasts were not significant (all $p > .298$). Thus, if at all, Affect seems to have a "protective" effect against potential increases in empathic resonance after the Perspective Module. As shown in the bottom panels (b-c and e-f), these effects were driven by changes in the emotional condition.

Figure S6. ToM Performance.

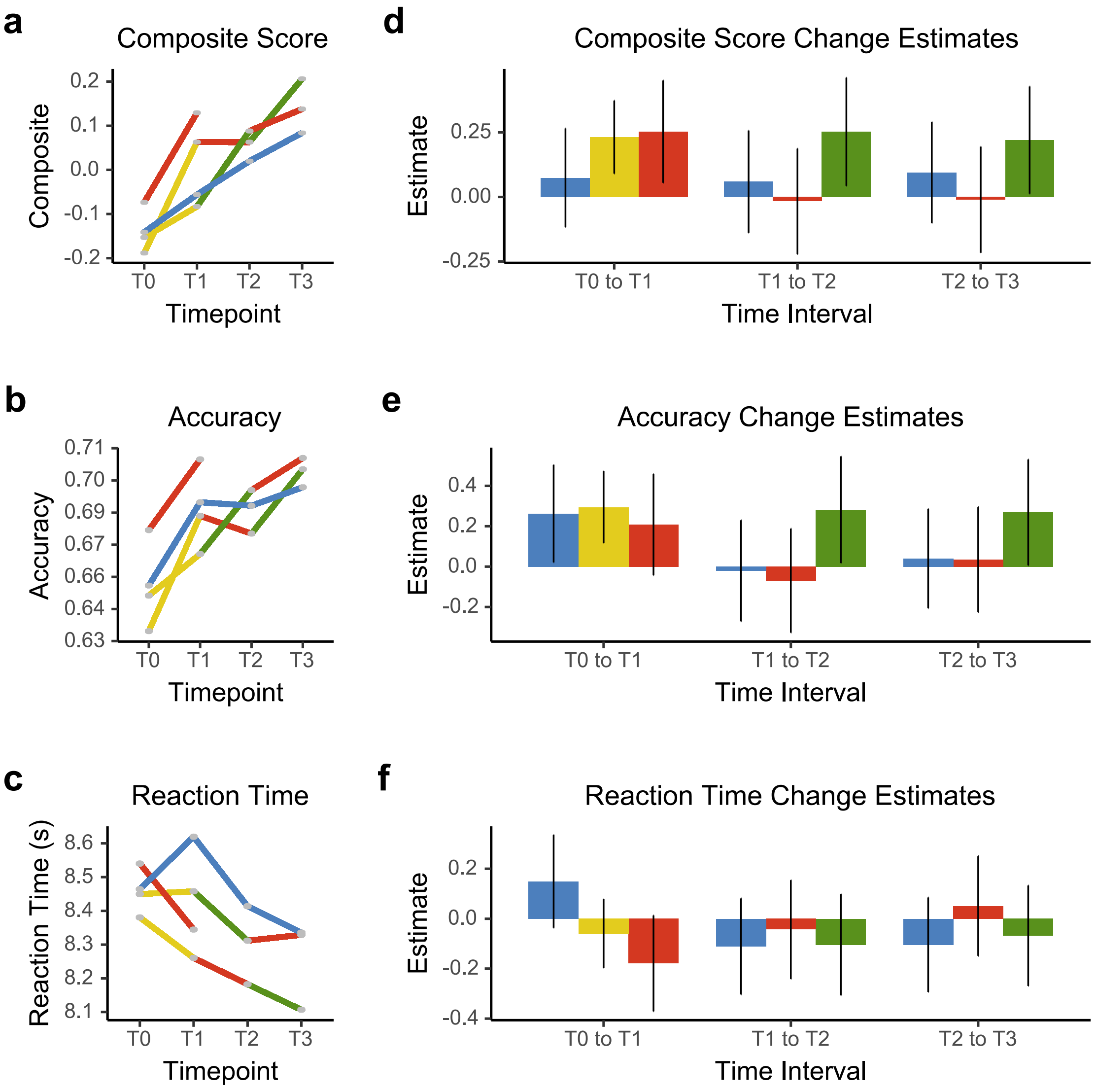

Fig. S6. Descriptive data and model estimates for ToM performance. Scores represent performance in the multiple choice questions following ToM videos. Panels on the left show descriptive plots of mean values for (**a**) composite scores of accuracy and reaction time, (**b**) accuracy (**c**) reaction time. The mean values of each individual were used to calculate change scores for each available pair of two consecutive time points, which were used to estimate effects for retest and the three training modules shown in (**f-h**). Error bars indicate 95% confidence intervals. See the main text for details on statistical tests.

References

Singer, T., Kok, B. E., Bornemann, B., Zurborg, S., Bolz, M., & Bochow, C. A. (2016). *The ReSource Project. Background, Design, Samples, and Measurements (2nd ed.)*. Leipzig: Max Planck Institute for Human Cognitive and Brain Sciences.

Trautwein, F.-M., Singer, T., & Kanske, P. (2016). Stimulus-Driven Reorienting Impairs Executive Control of Attention: Evidence for a Common Bottleneck in Anterior Insula. *Cerebral Cortex, 26*(11), 4136-4147.
